# A Core Outcome Set for Stillbirth Care: An International Consensus Study

**DOI:** 10.1111/1471-0528.18265

**Published:** 2025-07-07

**Authors:** Danya Bakhbakhi, Anna Davies, Dimitrios Siassakos, Lisa Hinton, James M. N. Duffy, Heatherjane Dangerfield, Maggie Redshaw, Abi Merriel, Amy Howell, Alexander E. P. Heazell, Christy Burden, Abigail Fraser, iCHOOSE Collaborative and Patient & Public Involvement Groups, Vicki Flenady, Soo Downe, Pauline Slade, Aleena Wojcieszek, Heloisa de Oliveira Salgado, Lindsey Wimmer, Danielle Pollock, Neelam Aggarwal, Susannah Hopkins Leisher, Mehali Patel, Vicki Flenady, Soo Downe, Pauline Slade, Aleena Wojcieszek, Heloisa de Oliveira Salgado, Lindsey Wimmer, Danielle Pollock, Neelam Aggarwal, Susannah Hopkins Leisher

**Affiliations:** ^1^ Translational Health Sciences, Bristol Medical School University of Bristol Bristol UK; ^2^ Institute for Women's Health, University College London London UK; ^3^ Nuffield Department of Primary Care Health Sciences University of Oxford Oxford UK; ^4^ Centre for Reproductive Medicine St Bartholomew's Hospital London UK; ^5^ Sands and iCHOOSE Parent and Public Representative Cardiff UK; ^6^ Brazelton Centre Cambridge UK; ^7^ Department of Women's and Children's Health University of Liverpool Liverpool UK; ^8^ Maternal and Fetal Health Research Centre, Faculty of Biology, Medicine and Health University of Manchester Manchester UK; ^9^ Population Health Sciences, Bristol Medical School University of Bristol Bristol UK

**Keywords:** consensus development study, core outcome set, core outcomes, outcome reporting bias, stillbirth

## Abstract

**Objective:**

To develop a core outcome set for stillbirth care.

**Design:**

Consensus development study.

**Setting:**

International.

**Population:**

542 participants from 29 countries, including 381 parents or family members who have experienced stillbirth, 192 care professionals and researchers (31 of which identified as both parent and professional). 95.6% of parents and 86.5% of professional stakeholders were from in high‐income countries.

**Methods:**

Modified Delphi method and consensus meetings.

**Results:**

Stakeholders agreed upon 8 core outcomes to measure in all stillbirth care studies; an additional 11 outcomes for specific interventions or care were also decided. Core outcomes for all stillbirth care studies were life‐threatening complications and maternal death, parents' experience of respectful and supportive care, grief, mental health and emotional wellbeing, isolation, stigma, impact on work, impact on relationship with immediate family. Outcomes for studies assessing interventions to understand the cause of stillbirth (investigations): cause of death identified and parents' understanding of the cause of death. Outcomes in studies assessing subsequent pregnancy after stillbirth: antenatal complications for mother, antenatal complications for baby, survival of baby, neonatal outcomes and attachment to baby. Outcomes for when a stillbirth occurs in a multiple pregnancy: survival of other baby/ies, preterm birth, pregnancy complications for baby/ies and neonatal outcomes.

**Conclusion:**

This core outcome set for stillbirth care can be used in future trials and systematic reviews to minimise outcome‐reporting bias, allow comparability of interventions in meta‐analyses and ultimately reduce research wastage.

## 1 Introduction

Following a stillbirth there are significant consequences for families including morbidity, mortality, psychological and social consequences [1]. There is a demonstrable need to improve bereavement care following a stillbirth. Most countries lack evidence‐based guidelines for the provision of care [2, 3]. The development of an international evidence base for effective stillbirth care interventions across the life‐course is, therefore, urgently required. This includes the immediate hospital care after a stillbirth is diagnosed, psychosocial support in the community, pre‐conception care and care in subsequent pregnancies and, potentially, for later life.

Well‐designed randomised and non‐randomised trials are needed to evaluate care after stillbirth. This has been identified as a top research priority by bereaved parents and care practitioners [4]. However, a systematic review of interventions after stillbirth identified significant research evidence gaps [5] and variation in outcome reporting. There was also a failure to include the perspectives of parents with lived experience of stillbirth both when designing care packages and in their evaluation [5].

In‐depth qualitative interviews found long‐term outcomes such as isolation, impact on work and outcomes in a subsequent pregnancy were important to bereaved parents [6]. Yet, studies evaluating stillbirth care have not consistently reported on these outcomes [5]. Moreover, the variation in, and inconsistent assessment of, the same outcome across studies restricts the ability to translate stillbirth care research into relevant evidence‐based practice and improvements in bereavement care [5, 7].

To address the lack of progress in stillbirth care research, our aim was to develop a core outcome set for stillbirth care research (iCHOOSE Study) [8]. A core outcome set is a minimum set of outcomes that should be reported in all clinical trials of health or health care, developed by robust consensus methods, involving key stakeholders such as patients, healthcare professionals and researchers in the outcome selection [9]. Core outcome sets have been advocated by journals and trial funders to help improve evidence synthesis in meta‐analyses and allow comparability between trials [9]. Several core outcome sets in Obstetrics and Gynaecology have been successfully developed with stakeholder engagement to minimise potential research wastage [10, 11, 12, 13].

## 2 Methods

This study was prospectively registered with the Core Outcome Measures in Effectiveness Trials (COMET) Initiative (registration number 775 [14]), developed with reference to the Core Outcome Set‐STAndards for Development (COS‐STAD) and reported using the Core Outcome Set‐STAndards for Reporting as reference [15, 16] (See Appendix S1). A protocol with pre‐defined scope and consensus methods was published [8].

In brief, the scope of this study was to develop a minimum set of outcomes that are relevant to families who have experienced a stillbirth from any cause in a singleton or multiple pregnancy. The interventions covered include the medical and psychological care that parents (and families) receive after a stillbirth has been identified and in a subsequent pregnancy. We aimed for this core outcome set to be applicable to all high‐income, middle‐income and low‐income countries. Our objective was to develop a core outcome set that could be used in all stillbirth care effectiveness research (e.g., randomised controlled trials, observational studies and systematic reviews). Furthermore, it could be used in implementation studies and the evaluation of clinical practice guidelines, care pathways for bereaved parents and training for healthcare professionals.

An international steering committee was established to inform methodological decisions, including: parents and family members with lived experience of stillbirth, care professionals (including obstetricians, midwives and health psychologists) and stillbirth researchers from Africa, Australia, Europe, North America, South America and Asia. The core outcome set development process was informed by current methodological consensus practice as recommended by the COMET initiative and relevant published literature [9].

### 2.1 Identification of Outcomes and Delphi Survey Development

**FIGURE 1:**
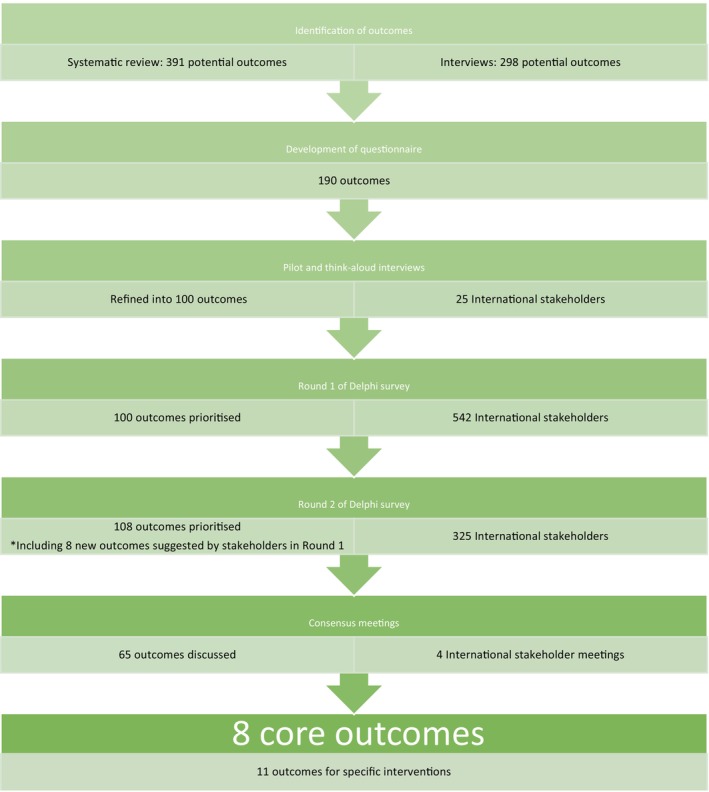
Stages of outcome selection for stillbirth care research core outcome set.

The core outcome set development process is presented in Appendix S2 Figure 1. Potential outcomes were identified from a systematic review and analysis of in‐depth interviews with 40 parents and family members with lived experience of stillbirth [5, 6]. A comprehensive outcomes inventory with plain‐language definitions was developed. This was piloted using iterative think‐aloud interviews with 26 international stakeholders and input from the steering committee [8, 17]. The outcomes inventory was inputted into REDCap (Research Electronic Data Capture) [18]. Due to resource limitations, the survey was developed in the English language only. A Delphi method, adapted to the study, was followed, including a two‐round survey and four consensus meetings to decide the core outcome set [8].

### 2.2 Recruitment

A diverse international sample of stakeholders (parents and family members with a lived experience of stillbirth, researchers and care professionals) was invited to participate in the two‐round survey through an advert containing a web link to the Delphi survey. Stakeholders were recruited through a network of professional contacts, support groups, social media, organisations such as the International Stillbirth Alliance, Sands, Centre of Research Excellence in Stillbirth (CRE), Stillbirth Foundation Australia, Star Legacy Foundation, Twins Trust and Still a Mum Africa, and by contacting published stillbirth researchers identified from the previous systematic review [5].

The study aimed to recruit 100 participants per stakeholder group (parents and professionals) anticipating a 20% attrition rate. The sample size for the modified Delphi method does not rely on statistical power and the estimated size was based on previous core outcome set development studies and guidelines (sample size 13–222) [7, 12, 13, 14, 19, 20].

### 2.3 Round 1 of Delphi

Prior to participating, stakeholders were presented with a research information leaflet and an explanatory video (https://www.youtube.com/watch?v=R8up4now2vM). Written informed consent was obtained from all participants. In Round 1, participants scored outcomes using a nine‐point Likert scale, scoring one to three (limited importance), four to six (important not critical) and seven to nine (critical). The scale was devised by the Grading of Recommendations Assessment, Development and Evaluation (GRADE) working group, which has been utilised for core outcome set development [21]. Participants were given the opportunity to suggest additional outcomes at the end of the survey, to be incorporated into the next round. Participants could select ‘unable to rate’ if they felt they did not have relevant expertise to rate an outcome.

### 2.4 Round 2 of Delphi Survey

In Round 2, each participant received their previous scores for Round 1, the median scores for each stakeholder group (described as the ‘average’), and histograms summarising the distribution of scores from Round 1 (one graph for parents and one for professionals). Participants were asked to reflect on their own score having been given the summary results of each stakeholder group from Round 1, before re‐scoring each outcome. Eight new outcomes were entered into Round 2 as suggested by participants from Round 1.

### 2.5 Consensus Definition

After Round 2, a standardised consensus definition was applied to enable core outcomes to be identified for discussion in the consensus meetings. In the protocol, the plan was as follows:

1. ‘Consensus in’ (classify as a core outcome): Over 70% of participants in at least one stakeholder group score outcome ‘critical’ (score seven to nine) and less than 15% of participants in at least one stakeholder group score outcome ‘limited importance’ (score one to three);
2. ‘Consensus out’ (do not classify as a core outcome): Over 70% of participants in at least one stakeholder group score outcome ‘limited importance’ (score one to three) and less than 15% of participants in at least one stakeholder group score outcome ‘critical’ (score seven to nine);
3. or ‘No Consensus’ (do not classify as a core outcome): Anything else [7, 15].

This consensus definition has been advocated by COMET and used in previous core outcome set development [9]. However, the protocol also included the potential to implement a tighter ‘consensus in’ definition as a possible approach in case many outcomes were scored as critical [8]. In the event, using the planned definition meant that over 77 outcomes would have been included for consideration in the consensus meetings. Therefore, after Round 2 of the Delphi survey prior to the consensus meetings and after discussion with members of the steering committee, this was revised to a potential ‘core outcome’ being: over 75% of participants in at least one stakeholder group score outcome ‘critical’ (score seven to nine) and less than 15% of participants across all stakeholder groups score ‘limited importance’ (score one to three).

### 2.6 Analysis

Data were imported into IBM SPSS Statistics Version 28.0. Descriptive statistics were generated for the demographic data (*N* and %). Scores for each outcome were analysed according to stakeholder category: (1) parents or family members and (2) professionals. The count and percentage of each stakeholder group rating outcomes of limited importance (one to three), important not critical (four to six) and critical (seven to nine) were calculated and compared. Median scores were calculated, and histograms were produced for each outcome for the Round 2 survey.

### 2.7 Attrition Bias Analysis

Median scores and the percentage of stakeholders scoring an outcome as ‘critical’ (seven to nine) were compared between those completing both rounds versus Round 1 only. The Mann Whitney U test was applied to test for differences in scoring using a 5% significance level.

### 2.8 Consensus Meetings

Four meetings took place to agree on the final core outcome set: (1) A preliminary parents‐only meeting; then three meetings with all participants to determine: (2) The core outcome set for all stillbirth care (all participants); (3) Core outcomes relevant to subsequent pregnancy care and (4) Core outcomes relevant to stillbirth in multiple pregnancy. The first meeting took place with parents only, to allow them to voice their opinions without potentially feeling intimidated by the presence of care providers. Parents had the opportunity to vote on outcomes, the results of which were used to guide discussion in the subsequent meeting. All stakeholders were invited to participate in the consensus meetings at the end of Round 2 of the Delphi questionnaire. Meetings were held via Zoom teleconferencing software, with members of the steering committee facilitating (D.B., A.D., C.B., D.S., A.H.) and a representative from Sands present to support parents if required.

A modified Nominal Group Technique was used in the consensus meetings [22]. We aimed to recruit eight to ten participants from each stakeholder group, as this number has yielded useful results in the development of previous core outcome sets [10, 11, 23]. Prior to the meeting, attendees were sent an information pack containing the results of the Delphi survey. Informed consent was obtained for the meetings to be audio and video recorded. A facilitator (D.B.) presented the results from Round 2. All potential core outcomes reaching the standardised definition for ‘Consensus in’ outcomes were discussed. Merging of similar outcomes were proposed by the research team and stakeholders in the consensus meetings as a large number of outcomes were voted ‘Consensus in’. Participants were asked to contribute their opinions on the relative importance of outcomes and the feasibility of outcome measurement. Participants were able to reformulate outcomes to improve clarity and relevance. Participants were asked to vote on each outcome as: ‘Consensus in—outcome included in the final core outcome set’, ‘Consensus out—outcome not included in the final core outcome set’ or ‘No consensus—outcomes for which opinions on inclusion are divided’. Outcomes scored ‘Consensus in’ by 75% or more of participants were included in the core outcome set.

During the first all‐stakeholders meeting (meeting 2), a framework to organise outcomes was proposed as it was noted that it may not be feasible to measure all core outcomes in all circumstances. For example, measuring whether the cause of death was identified would not be suitable to measure in a study of psychological counselling. This outcome framework was based on the OMERACT Filter 2.1 [24], which organises outcomes into mandatory outcomes in all trials and mandatory outcomes in specific circumstances. At the end of the fourth meeting, the final core outcome set was agreed. The meetings were transcribed verbatim to document the meeting.

## 3 Results

A total of 240 stillbirth care research studies reported 391 outcomes and analysis of 29 interviews with parents with lived experience of stillbirth identified 298 additional outcomes [5, 6]. After reformulation and grouping of outcomes by two researchers (D.B. & A.D.), 190 outcomes were entered into a pilot think‐aloud survey. After further refinement, 100 unique outcomes were entered into the Delphi survey (see Figure 1).

A total of 542 stakeholders commenced the Delphi survey; 381 identified themselves as a parent or family member with lived experience of stillbirth and 192 as professionals (researchers or health care professionals). Thirty‐one participants identified themselves as both a parent or family member and a professional. Due to the recruitment approach, it was not possible to ascertain the total number of stakeholders invited to participate, and therefore, the response rate cannot be calculated. Stakeholders were from 29 countries and six continents (Africa, Asia, Australasia, Europe, North America and South America). See Table 1 for participant characteristics and Appendix S3 for more detailed information on country of participation and ethnicity.

**TABLE 1:** Demographic characteristics of participants.

Parents' demographics			Parents' demographics			Parents' stillbirth experience			Stakeholder profession		
Characteristic	Total number		Characteristic	Total number		Characteristic	Total number		Characteristic	Total number	
**Age**	***N***	**%**	**World Bank Lending Group** *	***N***	**%**	**When did stillbirth happen**	***N***	**%**	**Stakeholder profession**	***N***	**%**
Under 21	2	0.5	High Income Country	364	95.6	I don't know when the stillbirth occurred	14	3.8	Stillbirth researcher	54	28.1
21–30	48	12.6	Upper Middle‐Income Country	12	3.1	During labour or birth	57	15.4	Stillbirth charity representative	26	13.5
31–40	210	55.1	Lower Middle‐Income Country	3	0.5	Before labour and birth	299	80.8	Midwife	61	31.8
41–50	95	24.9	Lower Income Country	2	0.01	**Twin or multiple pregnancy**	***N***	**%**	Obstetric doctor	39	20.3
51–60	11	2.9	**Number of stillbirths**	***N***	**%**	Stillbirth in a twin/multiple pregnancy	17	4.6	General/family practitioner	4	2.1
Over 60	15	3.9	1	358	96.8	**Number of previous pregnancies**	***N***	**%**	Psychologist/counsellor	23	12
**Male**	15	3.9	2	9	2.4	0	181	48.9	Nurse working in stillbirth care	18	9.4
**Female**	365	96.1	3	2	0.4	1	88	23.8	Chaplain or other religious/spiritual	1	0.5
**Gender same as birth**	***N***	**%**	4	1	0.3	2	51	13.8	Stillbirth peer supporter	17	8.9
Yes	377	99	**Time since stillbirth**	***N***	**%**	3	24	6.5	Stillbirth advocate	16	8.3
No	3	0.8	In the last 6 months	57	15.4	4	9	2.4	Social worker	2	1
Prefer not to answer	1	0.3	6–12 months	36	9.7	> 4	17	4.6	Perinatal pathologist	1	0.5
**Employed full time**	206	49.6	1–5 years	143	38.6	**Older children in family**	***N***	**%**	Other	18	9.4
**Employed part time**	78	18.8	6–10 years	73	19.7	Yes	166	44.9	**Years held in position**	***N***	**%**
**Not currently employed**	25	6	11–15 years	27	7.3	No	204	55.1	Less than 5 years	55	28.6
**Retired**	10	2.4	16–20 years	14	3.8						
**Other**	14	3.4	More than 20 years ago	20	5.4	**Subsequent pregnancy after stillbirth**	138**	37.3	5–10 years	59	30.7
**Education**	***N***	**%**	**Gestation of recent stillbirth**	***N***	**%**	**Subsequent younger child**	190	51.4	11–20 years	45	23.4
Pre‐high school education (e.g., primary, elementary)	1	0.3	20–23 weeks	39	10.5	**None of these apply**	103	27.8	More than 20 years	33	17.2
High school education (e.g., secondary)	33	8.7	24–27 weeks	40	10.8	**Number of subsequent children**	***N***	**%**	**World Bank Lending Group** *	***N***	**%**
University or college education	266	69.8	28–31 weeks	36	9.7	1	116	62.7	High‐Income Country	166	86.5
Professional training	65	17.1	32–36 weeks	87	23.5	2	55	29.7	Upper Middle‐Income Country	7	3.6
Other	16	4.2	37–42 weeks	168	45.4	3	9	4.9	Lower Middle‐Income Country	10	5.2
			43–44 weeks	0	0	4	5	2.7	Lower Income Country	9	4.7

* Note some participants are accounted for twice as 31 participants were both parents and professionals.

** Note this number is could be higher as some participants who had subsequent children did not select subsequent pregnancy box in the Delphi survey.

Three hundred and twenty‐stakeholders participated in Round 2 of the Delphi survey. Round 2 was completed by 207 (55%) of parents or family members and 137 (70%) care professionals who completed Round 1. Appendix S4 presents a summary comparison of outcome voting results between parents and professionals scores in both Round 1 and 2 of the Delphi Survey. Following Round 2, 65 outcomes reached the threshold for ‘Consensus in’ based on the definition above.

### 3.1 Attrition Analysis

The overall attrition between Round 1 and Round 2 was 40%. The country of participation and score differences between those who completed Round 2 and those who did not are shown in Appendix S5. Participants who did not complete Round two of the Delphi had comparable median scores in Round 1, apart from two outcomes ‘Other mental health difficulties’ and ‘Mental health treatment’, which were scored as ‘critical’ by non‐completers of Round 2 (*p* < 0.05). This score difference did not impact on which outcomes were entered into the consensus meeting for discussion as both these outcomes were later deemed ‘critical’ in Round 2 of the Delphi.

### 3.2 Consensus Meeting

Consensus meetings were conducted in January (*n* = 15 participants), February (*n* = 31) and April 2022 (*n* = 19) and May 2023 (*n* = 14). Appendix S6 summarises participant characteristics. Appendix S7 gives voting scores and detailed illustrative quotes from the consensus meetings. Sixty‐five outcomes were considered in the consensus meetings. In addition, participants recommended and voted on the reformulation of five ‘consensus’ outcomes and re‐entered one additional ‘no consensus’ outcome (Stigma) into the process. Stakeholders agreed ‘Stigma’ was a demonstrable outcome and important outcome to measure as a Stigma Scale for stillbirth has been developed and validated [25]. Furthermore, the Stigma scale includes measurement of ‘Perceived opportunities to talk about stillbirth and baby’ an outcome which was deemed ‘critical’ in Round 2 of the Delphi.

### 3.3 Final Core Outcome Set

Following discussion and voting on each outcome, the final outcome set included 19 measures: 8 core outcomes and 11 additional ones, to be measured for studies of specific interventions or care. See Table 2 for the core outcome set and plain‐language definitions of outcomes. Table 3 illustrates how the core outcome set for stillbirth care could be utilised in research and clinical practice.

**TABLE 2:** Core outcome set and plain‐language definitions of outcomes.

Outcome	Plain‐language definition
**A. Core outcomes for stillbirth care research**	
Life‐threatening complications and death	Life‐threatening complications in parents, for example, organ failure, excessive blood loss requiring transfusion, shock, blood clots or a severe infection (sepsis), retained placenta, near‐misses for mortality and morbidity, complications that may require surgery or hospital readmission. The death of a parent while pregnant, during or following birth, including suicide
Parents' experience of respectful and supportive care	Parent's experience of care, before, during and after birth including experience of support, communication, perceived recognition of parenthood and shared decision‐making
Grief	Grief including overwhelming or complicated grief, coping and living with grief, feelings of self‐blame, guilt or failure, acknowledgement of grief
Mental health and emotional wellbeing	Mental health and wellbeing including depression, anxiety, PTSD, other mental health difficulties, mental functioning, mental health treatment, suicidal thoughts and emotional wellbeing.
Isolation	Social isolation, for example, feeling alone or abandoned, loneliness, isolation and avoidance
Stigma	Stigma, for example, perceived shame, discrimination and opportunities to talk about stillbirth and baby
Impact on work	Impact on occupation (paid or unpaid work), for example, impact on job, being able to do job, unemployment, change of career, parental leave, support for return to work
Impact on relationship with immediate family	Impact on relationship with immediate family, such as partner, family and children, for example, positive or negative relationship consequences and perceived support
**B. Core outcomes for studies of specific interventions or care**	
*B1. Investigation of stillbirth outcomes*	
Cause of death identified	Cause(s) of death from the medical investigations to understand why a stillbirth happened
Parents' understanding of the cause of death	Measurement of whether parents understand the cause(s) of death or that no cause of death was found, including understanding risk of recurrence and management in subsequent pregnancy
*B2. Subsequent pregnancy outcomes*	
Antenatal complications for mother	Antenatal complications for mother, for example, bleeding, obstetric cholestasis, low lying placenta, premature rupture of membranes, gestational diabetes, pre‐eclampsia
Antenatal complications for baby	Antenatal complications for baby, for example, growth restriction, genetic or congenital anomaly
Survival of baby	Baby survival outcomes, for example, live birth, miscarriage, stillbirth, termination of pregnancy, neonatal death of baby in a subsequent pregnancy
Neonatal outcomes	Newborn outcomes, for example, Apgar score, birth weight of baby, admission to neonatal intensive care unit, gestational age, resuscitation of baby
Attachment to baby	Attachment to baby during subsequent pregnancy after stillbirth, for example, perceived connection or bonding with new baby and acceptance of pregnancy
*B3. Outcomes for when a stillbirth occurs in multiple pregnancy*	
Survival of baby/ies	Baby survival outcomes, for example, live birth, miscarriage, stillbirth, termination of pregnancy, neonatal death of surviving baby/ies
Preterm birth	Number of surviving baby(ies) born before 37 weeks, 34 and 28 weeks
Pregnancy complications for baby	Complications that risk the life of the surviving baby(ies). For example, growth restriction, fetal anaemia, requiring intrauterine transfusion, premature labour
Neonatal outcomes	Newborn outcomes. For example, admission to neonatal intensive care unit, Apgar score, birth weight

**TABLE 3:** Illustrates how the core outcome set for stillbirth care could be utilised in research and clinical practice.

Example of intervention	What outcomes should be measured?	
	Core outcomes	Outcomes in specific circumstances
Evaluation of implementation of a hospital bereavement care pathway with staff training (including investigations to understand why a baby has died)	Life‐threatening medical complications and death Parents experience of respectful and supportive care Grief Mental health and emotional wellbeing Isolation Stigma Impact on work Impact on relationship with immediate family	Cause of death identified Parent's understanding of the cause of death
Evaluation of parental engagement in the perinatal mortality review process	Life‐threatening medical complications and death Parents experience of respectful and supportive care Grief Mental health and emotional wellbeing Isolation Stigma Impact on work Impact on relationship with immediate family	Cause of death identified Parent's understanding of the cause of death
Evaluation of counselling or psychological support for bereaved parents following a stillbirth	Life‐threatening medical complications and death Parents experience of respectful and supportive care Grief Mental health and emotional wellbeing Isolation Stigma Impact on work Impact on relationship with immediate family	
Evaluation of implementing a complex intervention in a subsequent pregnancy including continuity of care, additional ultrasound scans and specialist care	Life‐threatening medical complications and death Parents experience of respectful and supportive care Grief Mental health and emotional wellbeing Isolation Stigma Impact on work Impact on relationship with immediate family	Antenatal complications for mother Antenatal complications for baby Survival of baby Neonatal outcomes Attachment to baby during pregnancy

## 4 Discussion

### 4.1 Main Findings

We developed a core outcome set for use in stillbirth care research, with extensive input from national and international stakeholders using rigorous consensus methodology. The development of a minimum agreed set of outcomes represents a significant milestone to address inconsistent outcome collection and reporting in studies of care after stillbirth.

### 4.2 Strengths & Limitations

A diverse range of stakeholders was involved in the development of the core outcome set from the outset. A total of 542 participants from 29 countries took part in a Delphi consensus process to rationalise outcomes of importance, which exceeded our recruitment target of 200 stakeholders. The utilisation of an online Delphi platform and web‐conferencing software enabled a greater number of international participants, from a broader range of geographical locations, to prioritise outcomes.

An inclusive maximum variation approach was adopted, and a range of parental stillbirth experiences are represented within the Delphi panel, for example, parents who experienced stillbirth at varying gestational ages. However, 69.8% of participating parents had a university or college education (this is higher than the population average); thus, the generalisability of the core outcome set may be limited. Parents experienced stillbirth from as recent as within the last 6 months to more than 20 years ago. There were no specific instructions in our consensus process about considering outcomes' relevance by time since the stillbirth. However, we plan to assess timing of outcome measurement in future research.

Approximately twice as many parents took part as professionals. This appears to be a relatively high proportion of patients compared to other core outcome sets that have been developed, and so is a notable strength of the study [26]. Incorporating bereaved parents' views throughout the consensus process, through seeking parental views from the patient and public panel group, representation on the project steering committee and piloting of the Delphi questionnaire, is likely to have encouraged participation in this study. The outcome ‘Isolation’ was derived directly from qualitative interviews with bereaved parents and has now been selected as a core outcome, demonstrating the value of patient involvement in core outcome set development [6].

The aim was to develop an international core outcome set and people from 29 countries participated. However, 90% of stakeholders were from high‐income countries, which is a limitation, as parents living in low‐ and middle‐income countries might have prioritised different outcomes in the core outcome set. Furthermore, stakeholders who did not speak English were not included. A core outcome set for stillbirth care in Sub‐Saharan Africa and South Asia, has now been developed (since the development of this core outcome set in collaboration with the iCHOOSE team) [27]. Interestingly, two outcomes in this iCHOOSE core outcome set were selected by stakeholders in this study conducted in low‐and middle‐income countries (LMICs): ‘Mental Health and emotional wellbeing’ and ‘Parent's experience of care and support’. These outcomes could be used in an international meta‐analysis to compare interventions in the future. Three alternative core outcomes were also selected in the core outcome set for LMICs: ‘Social wellbeing’, ‘Obstetric fistula’ and ‘Type of stillbirth’ [27].

It was noted that the overall response rate for Round 2 was 60% which is comparable to other core outcome set studies [10]. Possible explanations for attrition could be due to Round 2 being open for a shorter period (4 weeks) and the large number of outcomes that needed to be re‐rated. One study of published core outcome set Delphi studies found that larger panel sizes and the number of items were associated with significantly lower response rates [28]. Indirect methods such as recruitment through social media and charity organisations may have contributed to the lower response rates [28]. Despite this, the attrition analysis seems to suggest that the scoring patterns were mostly comparable between those who completed and those who did not complete Round 2.

### 4.3 Interpretation

The scope of this core outcome set was broad as it was anticipated that it could be applicable to all care provided after stillbirth. This includes studies of hospital bereavement care, investigations to understand the cause of stillbirth, psychological follow‐up and care in a subsequent pregnancy. It became apparent in this core outcome set development process that a ‘one size fits all approach’ may not be suitable to ensure relevant important outcomes are included in a minimum dataset. An outcomes framework is therefore proposed with a core set of outcomes and additional measures that relate to specific interventions and circumstances. This is similar to the OMERACT Filter 2.1 Onion that has been used in rheumatology to organise outcomes in core outcome sets [24].

Many outcomes were initially deemed to be critical to measure in all stillbirth care research studies following Round 2 of the Delphi survey. As such, a deviation from the original protocol was required, since the consensus definition of what outcomes were ‘Consensus in’ needed to be more restrictive. This led to fewer outcomes being discussed and outcomes being combined in the consensus meeting. Other developers have had to adapt their definitions based on the large number of outcomes being deemed important following their Delphi surveys [29]. Uncertainty exists about the best methodology to use to develop a core outcome set and further research should be conducted to ascertain how different consensus definitions affect which outcomes are included within a core outcome set and optimal methods.

This study has identified a minimum set of outcomes, which should be measured in all stillbirth care research. It is important to note that this core outcome set is not restrictive, and additional outcomes can be measured. Researchers should seek to add outcomes as relevant to their scope and setting of their study to ensure relevant factors are measured. Some of the outcomes within the core outcome set are broad domains, for example: ‘Life‐threatening complications’ and ‘Impact on relationship with immediate family’. This consensus process has started to define such outcome domains (Table 2) by developing definitions based on qualitative research with stakeholders [6]. Further research is required, as the current definitions may be too vague for use in clinical trials. However, in the interim, this core outcome set could begin to ensure that the most important types of outcomes are being collected and reported.

A variety of types of outcomes are included within the core outcome set, for example, composite, patient‐reported experience and clinical outcomes. Some of these outcomes could be measured using a variety of different outcome measurement tools, which could make direct comparisons challenging. Similarly, specific or novel measurement tools may need to be developed, as no outcome measurement tool currently exists to measure the outcome in the stillbirth population. COSMIN (Consensus‐based Standards for the selection of health Measurement INstruments) has produced guidance to inform the selection of outcome measurement instruments in core outcome set research using consensus methodology, which will form the basis of future research [30, 31].

It is envisaged that this core outcome set will minimise reporting bias and reduce research wastage, based on the requirements of the Standard Protocol Items: Recommendations for Interventional Trials (SPIRIT) guidance, that encourages trialists to ascertain whether there is a core outcome set relevant to their trial and utilise it if one exists [32]. In addition, research funding bodies such as NIHR and the editors of over 50 journals related to women's and newborn's health support the use of a common set of key trial outcomes in research [33].

## 5 Conclusion

It is recommended that this core outcome set for stillbirth care be used in studies assessing interventions after stillbirth, including, but not limited to, comparative effectiveness trials and observational studies and quantitative systematic reviews. Once outcomes are defined and core outcome measurement tools are selected, comparisons in meta‐analyses will be a possibility and will avoid potential research wastage. It will facilitate ascertaining what care is best, improve clinical decision making, with the aim to improve long‐term recovery and wellbeing for bereaved families.

## Author Contributions

Idea was conceived by D.B. and D.S., study concept and design were by D.B., A.D., D.S., L.H., J.M.N.D., M.R., H.D., A.E.P.H., C.B. and A.F. Acquisition of data was by D.B. and A.D. Support with survey from A.D., D.S., C.B. and A.F. Analysis and interpretation of data was by D.B., A.D., A.H., C.B. and A.F. D.B. drafted the manuscript. Critical revision of the manuscript for important intellectual content was by D.B., A.D., D.S., L.H., J.M.N.D., A.H., M.R., H.D., A.M., A.E.P.H., C.B. and A.F.

## Ethics Statement

Ethical approval was obtained from the University of Bristol Faculty of Health Sciences Research Committee (FREC) Ref:116535.

## Conflicts of Interest

D.B. is Trainee Scientific Editor for BJOG. A.E.P.H. is the Director of the Tommy's Stillbirth Research Centre, University of Manchester, which receives grant funding for stillbirth research. D.S. receives grant funding for stillbirth research from multiple sources and has been executive editor for BJOG.

## Supporting information


Appendix S1.

Appendix S2.

Appendix S3.

Appendix S4.

Appendix S5.

Appendix S6.

Appendix S7.

## Data Availability

The data that support the findings of this study are available on request from the corresponding author. The data are not publicly available due to privacy or ethical restrictions.
