# Supplementary material for: A Core Outcome Set for Stillbirth Care: An International Consensus Study
Source: BJOG. 2025 Jul 7;132(13):2149–59. doi: 10.1111/1471-0528.18265 (PMC12592755; doi:10.1111/1471-0528.18265)
Supplement: Supplementary file 1 — Appendix S1. [file BJO-132-2149-s005.docx]

| **Section/Topic** | **Item No.** | **Checklist Item** | **Page number** |
| --- | --- | --- | --- |
| **Title/Abstract** | | | |
| Title | 1a | Identify in the title that the paper reports the development of a COS | 1 |
| Abstract | 1b | Provide a structured summary | 3 |
| **Introduction** | | | |
| Background and objectives | 2a | Describe the background and explain the rationale for developing a COS | 5 |
|  | 2b | Describe the specific objectives with reference to developing a COS | 5 & 6 |
| Scope | 3a | Describe the health condition (s) and population (s) covered by the COS. | 6 |
|  | 3b | Describe the intervention covered by the COS | 6 |
|  | 3c | Describe the setting (s) in which the COS is to be applied | 6 |
| **Methods** | | | |
| Protocol/Registry Entry | 4 | Indicate where the COS development protocol can be accessed if available and/or the study registration details | 6 |
| Participants | 5 | Describe the rationale for the stakeholder groups involved in the COS process, eligibility criteria for participants from each group, and a description of how the individuals involved were identified. | 6 & 7 |
| Information Sources | 6a | Describe the information sources used to identify an initial list of outcomes. | 6 |
|  | 6b | Describe how outcomes were dropped/combined, with reasons (if applicable) | 6, 10 & 11 |
| Consensus Process | 7 | Describe how the consensus process was undertaken. | 7-9 |
| Outcome Scoring | 8 | Describe how outcomes were scored and summarised | 7-9 |
| Consensus Definition | 9a | Describe the consensus definition. | 9 |
|  | 9b | Describe the procedure for determining how outcomes were included or excluded from consideration during the consensus process. | 9 |
| Ethics and Consent | 10 | Provide a statement regarding ethics and consent issues for the study. | 22 |
| **Results** | | | |
| Protocol deviations | 11 | Describe any changes from the protocol (if applicable), with reasons, and describe what impact these changed have on results | 8 |
| Participants | 12 | Present data on the number and relevant characteristics of the people involved at all stages of the COS. | 13  Supporting information 3 & 6 |
| Outcomes | 13a | List all outcomes considered at the start of the consensus process. | Supporting information 4 |
|  | 13b | Describe any new outcomes introduced and any outcomes dropped with reasons during the consensus process. | Supporting information 4 & 7 |
| COS | 14 | List all the outcomes in the final COS | 17 |
| **Discussion** | | | |
| Limitations | 15 | Discuss any limitations in the COS development | 18 & 19 |
| Conclusions | 16 | Provide an interpretation of the final COS in the context of other evidence and implications for future research | 20 & 21 |
| **Other information** | | | |
| Funding | 17 | Describe sources of funding/role of funders | 4 |
| Conflicts of interest | 18 | Describe any conflicts of interest within the study team and how these were managed. | 21 & 22 |
