# Supplementary figures and images for: A Core Outcome Set for Stillbirth Care: An International Consensus Study
Source: BJOG. 2025 Jul 7;132(13):2149–59. doi: 10.1111/1471-0528.18265 (PMC12592755; doi:10.1111/1471-0528.18265)

## Figure 1: Development process for core outcome set for stillbirth care research

##
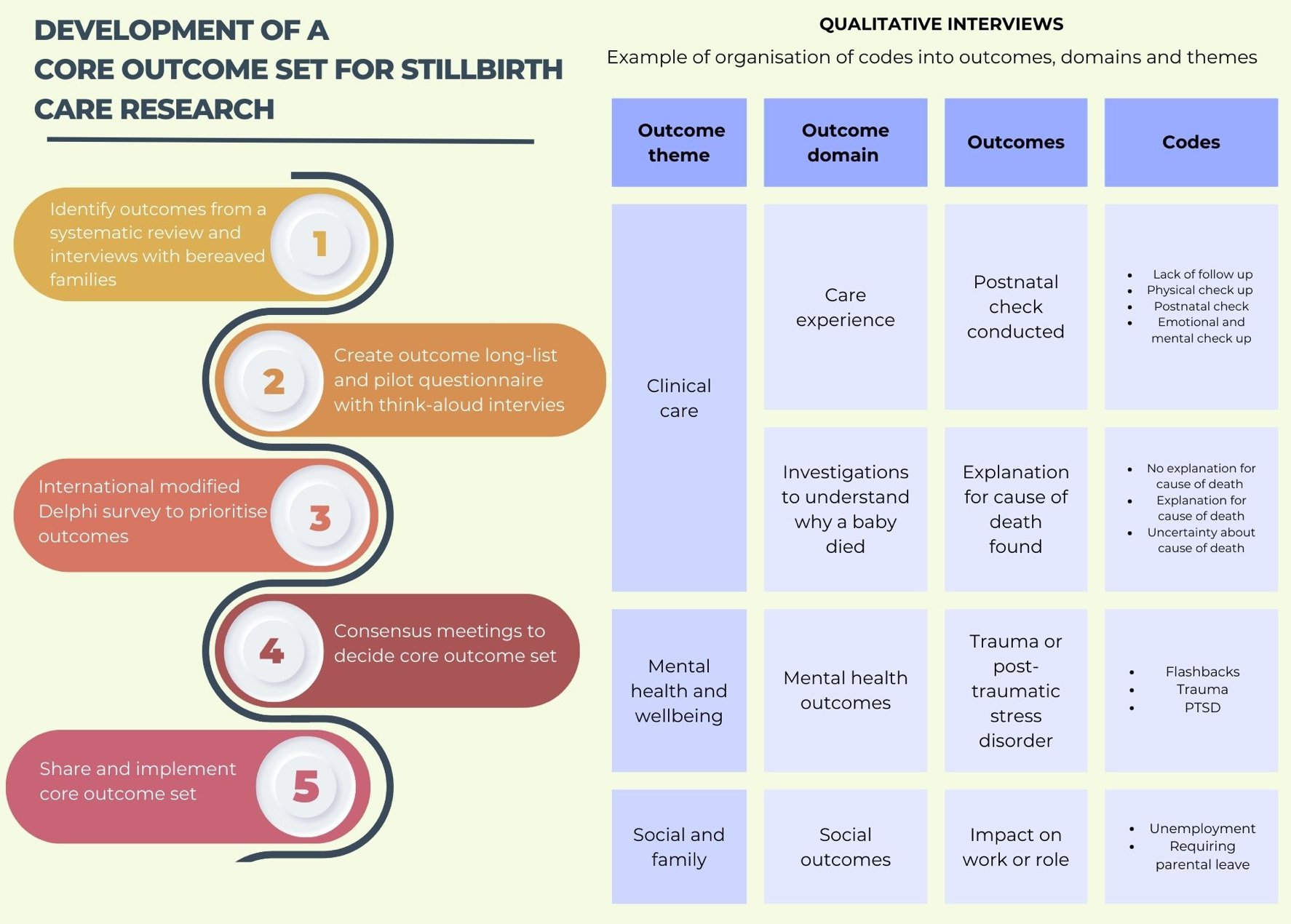

Supplement: Supplementary file 2 — Appendix S2. [file BJO-132-2149-s004.docx]
