# Supplementary material for: A Core Outcome Set for Stillbirth Care: An International Consensus Study
Source: BJOG. 2025 Jul 7;132(13):2149–59. doi: 10.1111/1471-0528.18265 (PMC12592755; doi:10.1111/1471-0528.18265)
Supplement: Supplementary file 3 — Appendix S3. [file BJO-132-2149-s003.docx]

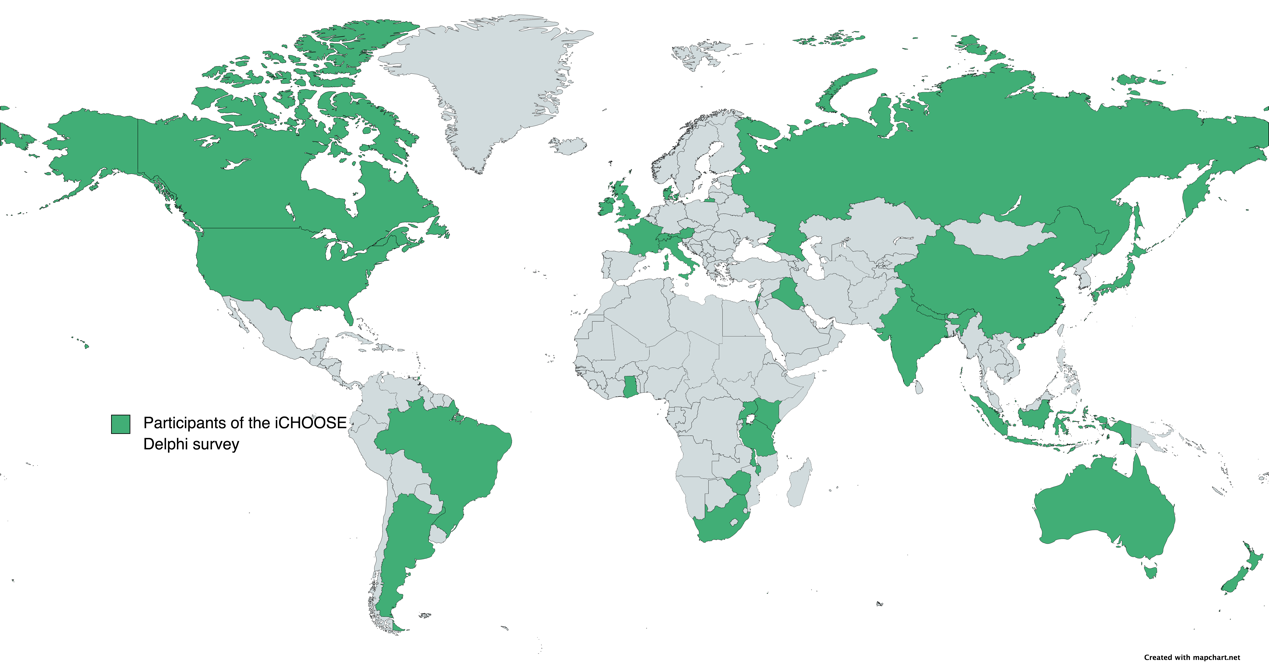


| **Country** | **World Bank Lending Group (Note 1)** | **Parent or family member** | | **Professional** | | **Total N by Country** |
| --- | --- | --- | --- | --- | --- | --- |
|  |  | N | % | N | % |  |
| Argentina | UMIC | 1 | 0.30% | 0 | 0.00% | 1 |
| Australia | HIC | 14 | 3.70% | 17 | 8.90% | 31 |
| Austria | HIC | 1 | 0.30% | 1 | 0.50% | 2 |
| Brazil | UMIC | 10 | 2.60% | 8 | 4.20% | 18 |
| Canada | HIC | 9 | 2.40% | 4 | 2.10% | 13 |
| China | HIC | 0 | 0.00% | 1 | 0.50% | 1 |
| Denmark | HIC | 0 | 0.00% | 2 | 1.00% | 2 |
| France | HIC | 1 | 0.30% | 1 | 0.50% | 2 |
| Ghana | LIC | 1 | 0.30% | 2 | 1.00% | 3 |
| India | LMIC | 1 | 0.30% | 2 | 1.00% | 3 |
| Indonesia | LMIC | 1 | 0.30% | 0 | 0.00% | 1 |
| Iraq | UMIC | 0 | 0.00% | 1 | 0.50% | 1 |
| Ireland {Republic} | HIC | 2 | 0.50% | 5 | 2.60% | 7 |
| Israel | HIC | 0 | 0.00% | 1 | 0.50% | 1 |
| Italy | HIC | 0 | 0.00% | 1 | 0.50% | 1 |
| Japan | HIC | 1 | 0.30% | 1 | 0.50% | 2 |
| Kenya | LMIC | 1 | 0.30% | 3 | 1.60% | 4 |
| Malawi | LIC | 0 | 0.00% | 2 | 1.00% | 2 |
| Nepal | LMIC | 0 | 0.00% | 2 | 1.00% | 2 |
| New Zealand | HIC | 0 | 0.00% | 2 | 1.00% | 2 |
| Russian Federation | HIC | 0 | 0.00% | 3 | 1.60% | 3 |
| South Africa | UMIC | 1 | 0.30% | 0 | 0.00% | 1 |
| Switzerland | HIC | 1 | 0.30% | 0 | 0.00% | 1 |
| Tanzania | LMIC | 0 | 0.00% | 1 | 0.50% | 1 |
| Trinidad & Tobago | HIC | 1 | 0.30% | 1 | 0.50% | 2 |
| Uganda | LIC | 1 | 0.30% | 3 | 1.60% | 4 |
| United Kingdom | HIC | 133 | 34.90% | 90 | 46.90% | 223 |
| United States | HIC | 201 | 52.80% | 36 | 18.80% | 237 |
| Zimbabwe | LMIC | 0 | 0.00% | 2 | 1.00% | 2 |
|  |  |  |  |  |  |  |

*Ethnicity of parents who participated in iCHOOSE Delphi survey (UK, Australia & USA)*

| **UK ethnicity of parents and family members** | **N** | **%** |
| --- | --- | --- |
| White British | 110 | 84.6 |
| White other | 12 | 9.2 |
| Mixed/multiple ethnic groups | 3 | 2.3 |
| Asian/Asian British | 3 | 2.3 |
| Black/African/Caribbean/Black British | 1 | 0.8 |
| Mixed | 1 | 0.8 |
| Total | 130 | 100 |
| **USA ethnicity/race** | **N** | **%** |
| White | 160 | 88.4 |
| Black or African American | 4 | 2.2 |
| Asian | 5 | 2.8 |
| Hispanic or Latino | 5 | 2.8 |
| Mixed | 7 | 3.9 |
| Total | 181 | 100 |
| **Australasia ethnicity** | **N** | **%** |
| Australian | 9 | 69.2 |
| Australian Aboriginal | 1 | 7.7 |
| British | 2 | 15.4 |
| Northern European | 1 | 7.7 |
| Total | 13 | 100 |
