# Supplementary material for: A Core Outcome Set for Stillbirth Care: An International Consensus Study
Source: BJOG. 2025 Jul 7;132(13):2149–59. doi: 10.1111/1471-0528.18265 (PMC12592755; doi:10.1111/1471-0528.18265)
Supplement: Supplementary file 4 — Appendix S4. [file BJO-132-2149-s002.docx]

| **Round 1 Delphi survey results** | | | | | | |
| --- | --- | --- | --- | --- | --- | --- |
| **Outcome** | **Parents or family member** | | | **Professionals** | | |
|  | **% Voted**  **7-9** | **Total who Voted on outcome** | **Total ‘unable to rate’ outcome** | **% Voted**  **7-9** | **Total who Voted on outcome** | **Total ‘unable to rate’ outcome** |
| **Labour and birth outcomes** | | | | | | |
| Induction of birth | 48.8% | 381 | 35 | 40.4% | 191 | 8 |
| Length of time from identification of a stillbirth to the birth | 60.8% | 381 | 11 | 44.6% | 192 | 6 |
| Pain relief for labour and birth | 54.2% | 381 | 10 | 51.4% | 192 | 7 |
| Type of birth | 57.1% | 381 | 10 | 53.8% | 192 | 6 |
| Complications during birth for mother or baby | 73.6% | 380 | 16 | 72.0% | 191 | 5 |
| **Postpartum maternal medical outcomes** | | | | | | |
| Maternal complications after birth | 67.3% | 368 | 10 | 68.3% | 187 | 4 |
| Maternal life-threatening complications after birth | 78.0% | 368 | 13 | 82.0% | 187 | 4 |
| Length of maternal hospital stay due to medical complications after a stillbirth | 45.3% | 368 | 8 | 40.4% | 187 | 4 |
| Maternal postpartum recovery | 67.3% | 368 | 4 | 52.7% | 187 | 3 |
| Maternal long term physical complications related to the birth | 57.7% | 368 | 9 | 60.3% | 187 | 3 |
| Maternal long term physical health outcomes | 58.9% | 366 | 11 | 54.3% | 187 | 3 |
| Maternal death | 83.4% | 366 | 23 | 85.2% | 186 | 4 |
| **Care experience outcomes** | | | | | | |
| Parents' experience of their care following stillbirth | 88.0% | 352 | 2 | 90.2% | 184 | 0 |
| Parents' experience of communication with care professionals | 89.1% | 352 | 2 | 90.2% | 184 | 0 |
| Parents' experience of support from care professionals | 91.7% | 352 | 2 | 90.2% | 184 | 0 |
| Perceived acknowledgement of parenthood by care professionals | 81.3% | 352 | 4 | 74.9% | 184 | 1 |
| Perceived acknowledgment of baby by care professionals | 86.8% | 352 | 4 | 82.0% | 184 | 1 |
| Impact of providing stillbirth care on healthcare professionals' | 79.3% | 352 | 4 | 64.5% | 184 | 1 |
| Formal complaints or legal action | 58.3% | 352 | 16 | 42.6% | 183 | 0 |
| **Investigation outcomes** | | | | | | |
| Uptake of medical investigations performed to understand why a baby died | 87.8% | 336 | 1 | 74.3% | 179 | 0 |
| Hospital review carried out by healthcare professionals to help understand why the baby died | 89.9% | 336 | 1 | 76.4% | 179 | 1 |
| Uptake of parental engagement in the hospital review to understand why a baby died | 80.1% | 336 | 5 | 63.3% | 179 | 2 |
| Cause of death identified | 92.8% | 336 | 3 | 74.9% | 179 | 0 |
| Parents' understanding of why their baby died | 90.1% | 336 | 2 | 79.9% | 179 | 0 |
| Parents' understanding about stillbirth | 89.0% | 336 | 1 | 75.8% | 178 | 0 |
| Improvements to care and patient safety | 92.8% | 336 | 3 | 83.1% | 178 | 0 |
| **Grief outcomes** | | | | | | |
| Overwhelming or complicated grief | 85.6% | 334 | 1 | 71.7% | 174 | 1 |
| Coping with grief | 85.6% | 334 | 1 | 69.2% | 174 | 2 |
| Feelings of self-blame, guilt or failure | 85.0% | 334 | 1 | 69.0% | 174 | 0 |
| Perceived acknowledgment of grief by others | 76.0% | 334 | 1 | 59.5% | 174 | 1 |
| Grief of whole family | 69.4% | 334 | 1 | 57.2% | 174 | 1 |
| **Mental health outcomes** | | | | | | |
| Depression | 78.0% | 330 | 2 | 75.0% | 173 | 1 |
|  |  |  |  |  |  |  |
| Anxiety | 79.9% | 330 | 1 | 70.3% | 173 | 1 |
| Post-traumatic stress disorder | 85.0% | 330 | 3 | 77.2% | 173 | 2 |
| Suicidal thoughts, attempted suicide, suicide | 83.3% | 330 | 6 | 84.3% | 173 | 1 |
| Other mental health difficulties | 74.2% | 330 | 5 | 70.8% | 173 | 2 |
| Drug and alcohol use | 65.9% | 330 | 7 | 63.7% | 173 | 2 |
| Mental functioning | 72.2% | 330 | 3 | 61.4% | 173 | 2 |
| Any mental health treatment (including type) | 74.0% | 329 | 2 | 63.4% | 173 | 1 |
| **Emotional outcomes** | | | | | | |
| Emotional wellbeing | 68.3% | 326 | 1 | 62.2% | 172 | 0 |
| Self esteem | 60.8% | 326 | 2 | 52.9% | 172 | 0 |
| Body confidence | 43.5% | 324 | 2 | 37.1% | 170 | 0 |
| Sense of control | 58.3% | 326 | 2 | 49.4% | 172 | 0 |
| Stress | 67.4% | 326 | 1 | 57.0% | 172 | 0 |
| Sexual wellbeing | 37.9% | 326 | 4 | 39.8% | 172 | 1 |
| **Whole person outcomes** | | | | | | |
| Impact on identity | 57.8% | 324 | 2 | 45.6% | 170 | 1 |
| Adjustment to new normal | 67.6% | 324 | 3 | 50.0% | 170 | 0 |
| Personal growth or positive impact | 46.9% | 324 | 2 | 39.6% | 170 | 1 |
| Impact on spirituality | 33.3% | 324 | 6 | 32.4% | 170 | 0 |
| Physical wellbeing | 59.0% | 324 | 2 | 50.6% | 170 | 0 |
| Quality of life | 62.5% | 324 | 1 | 64.1% | 170 | 0 |
| **Social outcomes** | | | | | | |
| Social impact | 56.5% | 319 | 2 | 49.7% | 170 | 1 |
| Opportunities to talk about stillbirth experience with others | 78.3% | 319 | 1 | 65.3% | 170 | 0 |
| Degree of isolation | 70.3% | 319 | 2 | 65.9% | 170 | 0 |
| Perceived stigma from community | 63.8% | 318 | 3 | 62.4% | 170 | 0 |
| Impact on work | 65.4% | 318 | 6 | 56.5% | 170 | 0 |
| **Relationship and support outcomes** | | | | | | |
| Impact on relationship with partner | 69.1% | 314 | 3 | 68.2% | 170 | 0 |
| Perceived support from partner | 65.6% | 314 | 3 | 61.2% | 170 | 0 |
| Impact on relationships with family, friends and community | 57.1% | 314 | 2 | 51.8% | 170 | 0 |
| Perceived support from family, friends and community | 56.2% | 314 | 1 | 49.4% | 170 | 0 |
| Perceived support with returning to work | 64.0% | 314 | 3 | 48.8% | 170 | 0 |
| Satisfaction with support resources and support groups | 59.9% | 314 | 2 | 57.4% | 170 | 1 |
| **Older children outcomes** | | | | | | |
| Psychological health of older children in the family | 77.0% | 154 | 6 | 53.9% | 170 | 3 |
| Impact on parenting | 79.5% | 154 | 3 | 56.8% | 170 | 1 |
| Support for older children and parenting | 74.3% | 154 | 6 | 48.5% | 170 | 1 |
| **Economic outcomes** | | | | | | |
| Financial costs for parents | 59.5% | 312 | 11 | 53.3% | 170 | 3 |
| Financial costs for health service and wider society | 43.0% | 312 | 10 | 42.5% | 170 | 3 |
| **Twin or multiple outcomes** | | | | | | |
| Survival of baby/ies after stillbirth is identified in a multiple pregnancy | 82.1% | 41 | 2 | 70.7% | 170 | 6 |
| Preterm birth of surviving baby(ies) after stillbirth is identified in a multiple pregnancy | 86.8% | 41 | 3 | 65.9% | 170 | 6 |
| Pregnancy complications that risk the life of the surviving baby(ies) after stillbirth is identified in a multiple pregnancy | 92.5% | 41 | 1 | 72.9% | 170 | 4 |
| Pregnancy complications for the mother after stillbirth is identified in a multiple pregnancy | 89.7% | 41 | 2 | 71.9% | 170 | 3 |
| Neonatal outcomes of surviving baby(ies) after stillbirth is identified in a multiple pregnancy | 79.5% | 41 | 2 | 74.3% | 170 | 3 |
| Neurodevelopment of surviving baby(ies) after stillbirth is identified in a multiple pregnancy | 71.8% | 41 | 2 | 62.7% | 170 | 4 |
| Medical health of surviving baby(ies) after stillbirth is identified in a multiple pregnancy | 71.8% | 41 | 2 | 63.5% | 170 | 3 |
| Attachment to surviving baby(ies) after stillbirth is identified in a multiple pregnancy | 70.0% | 41 | 1 | 60.5% | 170 | 3 |
| Psychological health of surviving child after stillbirth is identified in a multiple pregnancy | 55.0% | 41 | 1 | 52.4% | 170 | 2 |
| **Planning subsequent pregnancy outcomes** | | | | | | |
| Perceived support for planning next pregnancy after stillbirth | 84.4% | 312 | 4 | 69.4% | 170 | 0 |
| Need for fertility treatment after stillbirth | 59.7% | 312 | 17 | 38.5% | 170 | 1 |
| Infertility | 62.0% | 312 | 17 | 38.2% | 170 | 0 |
| Parents choosing not to become pregnant again after a stillbirth | 48.2% | 312 | 11 | 46.5% | 170 | 0 |
| **Subsequent pregnancy outcomes** | | | | | | |
| Time between stillbirth and next pregnancy | 52.6% | 138 | 1 | 41.2% | 170 | 0 |
| Number of pregnancies between stillbirth and live birth | 61.0% | 138 | 2 | 39.6% | 170 | 1 |
| Complications for the baby in a subsequent pregnancy after stillbirth | 92.6% | 138 | 3 | 73.4% | 170 | 1 |
| Complications for the mother in a subsequent pregnancy after stillbirth | 89.7% | 138 | 2 | 72.9% | 170 | 0 |
| Preterm birth in a subsequent pregnancy after stillbirth | 77.6% | 138 | 4 | 64.9% | 170 | 2 |
| Induction of labour in a subsequent pregnancy after stillbirth | 65.0% | 138 | 1 | 47.9% | 170 | 1 |
| Type of birth in a subsequent pregnancy after stillbirth | 58.4% | 138 | 1 | 47.3% | 170 | 1 |
| Birth and postpartum complications in a subsequent pregnancy after stillbirth | 71.5% | 138 | 1 | 56.2% | 170 | 1 |
| Survival of baby in a subsequent pregnancy after stillbirth | 91.2% | 138 | 2 | 77.1% | 170 | 0 |
| Newborn outcomes in baby born after stillbirth | 80.1% | 138 | 2 | 63.9% | 170 | 1 |
| Additional scans and clinic appointments during subsequent pregnancy after stillbirth | 85.4% | 138 | 1 | 63.9% | 170 | 1 |
| Unplanned hospital admission prior to birth of baby in a subsequent pregnancy after stillbirth | 79.6% | 138 | 1 | 51.8% | 170 | 0 |
| Perceived support for subsequent pregnancy, birth and parenthood after stillbirth | 74.5% | 138 | 1 | 60.0% | 170 | 0 |
| Coping in a subsequent pregnancy after stillbirth | 86.9% | 138 | 1 | 72.9% | 170 | 0 |
| Attachment to baby during subsequent pregnancy after stillbirth | 77.8% | 138 | 3 | 63.5% | 170 | 0 |
| Parents' satisfaction with care in a subsequent pregnancy after stillbirth | 78.4% | 138 | 4 | 61.8% | 170 | 0 |
| **Subsequent children outcomes** | | | | | | |
| Development of child born after a stillbirth | 46.3% | 167 | 3 | 32.7% | 170 | 2 |
| Medical health of child born after stillbirth | 49.1% | 167 | 4 | 34.5% | 170 | 2 |
| Attachment to a child born after stillbirth | 62.8% | 167 | 3 | 52.4% | 170 | 0 |
| Psychological health of child born after stillbirth | 52.4% | 167 | 3 | 45.3% | 170 | 0 |

| **Round 2 Delphi survey results** | | | | | | | |
| --- | --- | --- | --- | --- | --- | --- | --- |
| **Outcome** | **Parents or family member** | | | **Professionals** | | | **Notes** |
|  | **% Voted**  **7-9** | **Total who Voted on outcome** | **Total ‘unable to rate’ outcome** | **% Voted**  **7-9** | **Total who Voted on outcome** | **Total ‘unable to rate’ outcome** |  |
| **Labour and birth outcomes** | | | | | | | |
| Induction of birth | 36.60% | 207 | 21 | 26.50% | 137 | 5 |  |
| Length of time from identification of a stillbirth to the birth | 61.5% | 206 | 6 | 47.0% | 136 | 4 |  |
| Pain relief for labour and birth | 65.50% | 205 | 8 | 57.90% | 136 | 3 |  |
| Type of birth | 65.80% | 205 | 6 | 66.70% | 136 | 4 |  |
| Complications during birth for mother or baby | 83.20% | 205 | 9 | 88.80% | 136 | 2 | Both scores increased R2 >75% |
| **Postpartum medical outcomes** | | | | | | | |
| Maternal complications after birth | 80.60% | 196 | 5 | 81.10% | 134 | 2 | Both scores increased R2 >75% |
| Maternal life-threatening complications after birth | 92.40% | 196 | 8 | 88.30% | 134 | 2 |  |
| Length of maternal hospital stay due to medical complications after a stillbirth | 40.00% | 196 | 6 | 28.60% | 134 | 1 |  |
| Maternal postpartum recovery | 74.60% | 196 | 3 | 64.10% | 134 | 3 |  |
| Maternal long term physical complications related to the birth | 71.40% | 196 | 4 | 77.90% | 134 | 3 | Professionals score increased in R2 >75% |
| Maternal long term physical health outcomes | 70.40% | 196 | 7 | 71.80% | 134 | 3 |  |
| Maternal death | 93.60% | 196 | 8 | 94.70% | 134 | 1 |  |
| **Care experience outcomes** | | | | | | | |
| Parents' experience of their care following stillbirth | 93.70% | 193 | 2 | 96.90% | 131 | 1 |  |
| Parents' experience of communication with care professionals | 96.40% | 193 | 1 | 95.40% | 131 | 1 |  |
| Parents' experience of support from care professionals | 95.80% | 193 | 1 | 96.90% | 131 | 0 |  |
| ***New outcome*** Parents’ experience of shared decision making | 93.10% | 193 | 2 | 93.10% | 131 | 0 |  |
| Perceived acknowledgement of parenthood by care professionals | 90.00% | 193 | 3 | 91.50% | 131 | 1 | Professionals score increased in R2 >75% |
| Perceived acknowledgment of baby by care professionals | 93.70% | 193 | 3 | 91.60% | 131 | 0 |  |
| Impact of providing stillbirth care on healthcare professionals' | 88.50% | 193 | 2 | 80.20% | 131 | 0 | Professionals score increased in R2 >75% |
| ***New outcome*** Trust in healthcare professionals | 89.10% | 193 | 1 | 87.80% | 131 | 0 |  |
| Formal complaints or legal action | 60.10% | 193 | 10 | 48.50% | 131 | 1 |  |
| **Investigation outcomes** | | | | | | | |
| Uptake of medical investigations performed to understand why a baby died | 93.60% | 189 | 1 | 87.70% | 130 | 0 | Professionals score increased in R2 >75% |
| Hospital review carried out by healthcare professionals to help understand why the baby died | 95.20% | 189 | 3 | 88.40% | 130 | 1 |  |
| Uptake of parental engagement in the hospital review to understand why a baby died | 89.20% | 189 | 4 | 78.50% | 130 | 0 | Professionals score increased in R2 >75% |
| Cause of death identified | 95.70% | 189 | 1 | 92.30% | 130 | 0 | Professionals score increased in R2 >75% |
| Parents' understanding of why their baby died | 93.60% | 189 | 1 | 94.60% | 130 | 0 |  |
| Parents' understanding about stillbirth | 94.70% | 189 | 2 | 93.80% | 130 | 0 |  |
| ***New outcome*** Additional counselling or subsequent pregnancy care advice following investigations | 95.70% | 189 | 1 | 89.20% | 130 | 0 |  |
| ***New outcome*** Length of time investigations into understanding why a baby died continue after a stillbirth | 81.70% | 189 | 3 | 73.10% | 130 | 0 |  |
| Improvements to care and patient safety | 96.30% | 189 | 2 | 93.10% | 130 | 0 |  |
| **Grief outcomes** | | | | | | | |
| Overwhelming or complicated grief | 94.60% | 186 | 1 | 81.40% | 129 | 0 | Professionals score increased in R2 >75% |
| Coping with grief | 93.50% | 186 | 1 | 80.60% | 129 | 0 | Professionals score increased in R2 >75% |
| Feelings of self-blame, guilt or failure | 91.40% | 186 | 1 | 79.10% | 129 | 0 | Professionals score increased in R2 >75% |
| Perceived acknowledgment of grief by others | 87.40% | 185 | 2 | 77.50% | 129 | 0 | Professionals score increased in R2 >75% |
| Grief of whole family | 80.50% | 186 | 1 | 71.90% | 129 | 1 | Parents score increased in R2 >75% |
| **Mental health outcomes** | | | | | | | |
| Depression | 87.40% | 183 | 1 | 82.70% | 127 | 0 |  |
| Anxiety | 87.90% | 183 | 1 | 78.70% | 127 | 0 | Professionals score increased in R2 >75% |
| Post-traumatic stress disorder | 93.90% | 183 | 3 | 86.40% | 127 | 2 |  |
| Suicidal thoughts, attempted suicide, suicide | 91.60% | 183 | 4 | 90.50% | 127 | 1 |  |
| Other mental health difficulties | 84.50% | 183 | 2 | 77.2% | 127 | 0 | Professionals score increased in R2 >75% |
| Drug and alcohol use | 71.50% | 183 | 4 | 70.60% | 127 | 1 |  |
| Mental functioning | 81.70% | 183 | 3 | 73.60% | 127 | 2 | Parents score increased in R2 >75% |
| Any mental health treatment (including type) | 84.00% | 183 | 2 | 72.40% | 127 | 0 | Professionals score increased in R2 >75% |
| **Emotional outcomes** | | | | | | | |
| Emotional wellbeing | 80.30% | 179 | 1 | 72.60% | 124 | 0 | Parents score increased in R2 >75% |
| ***New outcome*** Self-compassion | 71.30% | 179 | 1 | 59.70% | 124 | 0 |  |
| Self esteem | 71.90% | 179 | 1 | 66.10% | 124 | 0 |  |
| Body confidence | 35.00% | 179 | 2 | 26.60% | 124 | 0 |  |
| Sense of control | 64.40% | 179 | 2 | 59.70% | 124 | 0 |  |
| Stress | 74.70% | 179 | 1 | 67.70% | 124 | 0 |  |
| Sexual wellbeing | 29.50% | 179 | 3 | 25.20% | 124 | 1 |  |
| *New outcome* Mindfulness | 46.90% | 179 | 2 | 35.80% | 124 | 4 |  |
| **Whole person outcomes** | | | | | | | |
| Impact on identity | 63.20% | 177 | 3 | 46.00% | 124 | 0 |  |
| Adjustment to new normal | 70.90% | 177 | 2 | 51.60% | 124 | 0 |  |
| Personal growth or positive impact | 35.60% | 176 | 2 | 27.40% | 124 | 0 |  |
| Impact on spirituality | 18.60% | 176 | 4 | 22.60% | 124 | 0 |  |
| Physical wellbeing | 65.50% | 176 | 2 | 62.10% | 124 | 0 |  |
| Quality of life | 70.70% | 176 | 2 | 71.80% | 124 | 0 |  |
| **Social outcomes** | | | | | | | |
| Social impact | 64.40% | 175 | 1 | 48.40% | 124 | 0 |  |
| Opportunities to talk about stillbirth experience with others | 85.60% | 175 | 1 | 75.00% | 124 | 0 | Professionals score increased in R2 >75% |
| Degree of isolation | 82.10% | 175 | 2 | 71.80% | 124 | 0 | Parents score increased in R2 >75% |
| Perceived stigma from community | 71.50% | 175 | 3 | 74.20% | 124 | 0 |  |
| Impact on work | 75.30% | 175 | 1 | 64.50% | 124 | 0 | Parents score increased in R2 >75% |
| **Relationship and support outcomes** | | | | | | | |
| Impact on relationship with partner | 78.50% | 174 | 2 | 79.70% | 123 | 0 | Both scores  increased R2  >75% |
| Perceived support from partner | 76.90% | 174 | 1 | 69.90% | 123 | 0 | Parents score increased in R2 >75% |
| Impact on relationships with family, friends and community | 67.10% | 174 | 1 | 68.30% | 123 | 0 |  |
| Perceived support from family, friends and community | 61.80% | 174 | 1 | 55.30% | 123 | 0 |  |
| Perceived support with returning to work | 68.80% | 174 | 1 | 56.90% | 123 | 0 |  |
| Satisfaction with support resources and support groups | 69.40% | 174 | 1 | 64.20% | 123 | 0 |  |
| **Older children outcomes** | | | | | | | |
| Psychological health of older children in the family | 88.10% | 85 | 1 | 68.00% | 122 | 0 |  |
| Impact on parenting | 86.70% | 85 | 2 | 71.90% | 122 | 1 |  |
| Support for older children and parenting | 77.40% | 85 | 1 | 62.30% | 122 | 0 | Parents score increased in R2 >75% |
| **Economic outcomes** | | | | | | | |
| Financial costs for parents | 57.40% | 174 | 5 | 61.20% | 122 | 1 |  |
| Financial costs for health service and wider society | 32.70% | 174 | 6 | 41.30% | 122 | 1 |  |
| **Twin or multiple outcomes** | | | | | | | |
| Survival of baby/ies after stillbirth is identified in a multiple pregnancy | 92.60% | 29 | 2 | 81.50% | 122 | 3 | Professionals score increased in R2 >75% |
| Preterm birth of surviving baby(ies) after stillbirth is identified in a multiple pregnancy | 92.30% | 29 | 3 | 75.60% | 122 | 3 | Professionals score increased in R2 >75% |
| Pregnancy complications that risk the life of the surviving baby(ies) after stillbirth is identified in a multiple pregnancy | 96.40% | 29 | 1 | 80.30% | 122 | 0 | Professionals score increased in R2 >75% |
| Pregnancy complications for the mother after stillbirth is identified in a multiple pregnancy | 92.60% | 29 | 2 | 77.90% | 122 | 0 | Professionals score increased in R2 >75% |
| Neonatal outcomes of surviving baby(ies) after stillbirth is identified in a multiple pregnancy | 96.30% | 29 | 2 | 82.00% | 122 | 0 | Professionals score increased in R2 >75% |
| Neurodevelopment of surviving baby(ies) after stillbirth is identified in a multiple pregnancy | 96.30% | 29 | 2 | 75.20% | 122 | 1 | Both scores increased R2 >75% |
| Medical health of surviving baby(ies) after stillbirth is identified in a multiple pregnancy | 92.60% | 29 | 2 | 71.30% | 122 | 0 | Parents score increased in R2 >75% |
| Attachment to surviving baby(ies) after stillbirth is identified in a multiple pregnancy | 78.60% | 29 | 1 | 75.20% | 122 | 1 | Both scores increased R2 >75% |
| Psychological health of surviving child after stillbirth is identified in a multiple pregnancy | 64.30% | 29 | 1 | 59.50% | 122 | 1 |  |
| **Planning subsequent pregnancy outcomes** | | | | | | | |
| Perceived support for planning next pregnancy after stillbirth | 87.10% | 172 | 2 | 81.10% | 122 | 0 | Professionals score increased in R2 >75% |
| Need for fertility treatment after stillbirth | 58.30% | 172 | 9 | 36.40% | 122 | 1 |  |
| Infertility | 58.50% | 172 | 8 | 37.20% | 122 | 1 |  |
| Parents choosing not to become pregnant again after a stillbirth | 36.00% | 172 | 11 | 37.20% | 122 | 1 |  |
| **Subsequent pregnancy outcomes** | | | | | | | |
| Time between stillbirth and next pregnancy | 51.20% | 127 | 2 | 44.60% | 121 | 0 |  |
| Number of pregnancies between stillbirth and live birth | 67.50% | 127 | 4 | 53.70% | 121 | 0 |  |
| ***New outcome*** Conception rate | 61.00% | 127 | 4 | 42.10% | 121 | 0 |  |
| Complications for the baby in a subsequent pregnancy after stillbirth | 94.40% | 127 | 2 | 81.00% | 121 | 0 | Professionals score increased in R2 >75% |
| Complications for the mother in a subsequent pregnancy after stillbirth | 92.80% | 127 | 2 | 79.30% | 121 | 0 | Professionals score increased in R2 >75% |
| Preterm birth in a subsequent pregnancy after stillbirth | 87.90% | 127 | 3 | 71.10% | 121 | 0 |  |
| Induction of labour in a subsequent pregnancy after stillbirth | 66.40% | 127 | 2 | 50.00% | 121 | 1 |  |
| Type of birth in a subsequent pregnancy after stillbirth | 51.20% | 127 | 2 | 43.30% | 121 | 1 |  |
| Birth and postpartum complications in a subsequent pregnancy after stillbirth | 78.90% | 127 | 4 | 60.80% | 121 | 1 | Parents score increased in R2 >75% |
| Survival of baby in a subsequent pregnancy after stillbirth | 95.20% | 127 | 3 | 89.20% | 121 | 1 |  |
| Newborn outcomes in baby born after stillbirth | 88.00% | 127 | 2 | 77.50% | 121 | 1 | Professionals score increased in R2 >75% |
| Additional scans and clinic appointments during subsequent pregnancy after stillbirth | 93.60% | 127 | 2 | 76.00% | 121 | 0 | Professionals score increased in R2 >75% |
| Unplanned hospital admission prior to birth of baby in a subsequent pregnancy after stillbirth | 82.30% | 127 | 3 | 60.30% | 121 | 0 |  |
| Perceived support for subsequent pregnancy, birth and parenthood after stillbirth | 88.00% | 127 | 2 | 76.00% | 121 | 0 | Professionals score increased in R2 >75% |
| Coping in a subsequent pregnancy after stillbirth | 92.00% | 127 | 2 | 86.00% | 121 | 0 | Professionals score increased in R2 >75% |
| ***New outcome*** Anxiety related to subsequent pregnancy and children | 92.80% | 127 | 2 | 82.60% | 121 | 0 |  |
| Attachment to baby during subsequent pregnancy after stillbirth | 87.30% | 127 | 1 | 78.50% | 121 | 0 | Professionals score increased in R2 >75% |
| Parents' satisfaction with care in a subsequent pregnancy after stillbirth | 88.80% | 127 | 2 | 79.30% | 121 | 0 | Professionals score increased in R2 >75% |
| **Subsequent children outcomes** | | | | | | | |
| Development of child born after a stillbirth | 28.00% | 95 | 2 | 13.30% | 120 | 0 |  |
| Medical health of child born after stillbirth | 31.50% | 95 | 3 | 19.20% | 120 | 0 |  |
| Attachment to a child born after stillbirth | 67.70% | 95 | 2 | 57.50% | 120 | 0 |  |
| Psychological health of child born after stillbirth | 52.70% | 95 | 2 | 38.30% | 120 | 0 |  |
