# Supplementary material for: A Core Outcome Set for Stillbirth Care: An International Consensus Study
Source: BJOG. 2025 Jul 7;132(13):2149–59. doi: 10.1111/1471-0528.18265 (PMC12592755; doi:10.1111/1471-0528.18265)
Supplement: Supplementary file 5 — Appendix S5. [file BJO-132-2149-s001.docx]

Comparison of country of participation of those who did and did not complete Round 2

| **Country** | | | | | |
| --- | --- | --- | --- | --- | --- |
|  | | Did not complete Round 2 | | Completed Round 2 | |
|  |  | N | % | N | % |
| Country | Argentina | 1 | 0.4 | 0 | 0.0 |
|  | Australia | 7 | 2.9 | 21 | 6.9 |
|  | Austria | 1 | 0.4 | 1 | 0.3 |
|  | Brazil | 10 | 4.2 | 7 | 2.3 |
|  | Canada | 5 | 2.1 | 7 | 2.3 |
|  | China | 1 | 0.4 | 0 | 0.0 |
|  | Denmark | 0 | 0.0 | 2 | 0.7 |
|  | France | 2 | 0.8 | 0 | 0.0 |
|  | Ghana | 0 | 0.0 | 2 | 0.7 |
|  | India | 1 | 0.4 | 2 | 0.7 |
|  | Indonesia | 1 | 0.4 | 0 | 0.0 |
|  | Iraq | 0 | 0.0 | 1 | 0.3 |
|  | Ireland {Republic} | 3 | 1.3 | 4 | 1.3 |
|  | Israel | 0 | 0.0 | 1 | 0.3 |
|  | Italy | 0 | 0.0 | 1 | 0.3 |
|  | Japan | 0 | 0.0 | 2 | 0.7 |
|  | Kenya | 2 | 0.8 | 1 | 0.3 |
|  | Malawi | 1 | 0.4 | 1 | 0.3 |
|  | Nepal | 1 | 0.4 | 1 | 0.3 |
|  | New Zealand | 1 | 0.4 | 1 | 0.3 |
|  | Russian Federation | 1 | 0.4 | 2 | 0.7 |
|  | South Africa | 1 | 0.4 | 0 | 0.0 |
|  | Switzerland | 0 | 0.0 | 1 | 0.3 |
|  | Tanzania | 0 | 0.0 | 1 | 0.3 |
|  | Trinidad & Tobago | 1 | 0.4 | 1 | 0.3 |
|  | Uganda | 2 | 0.8 | 2 | 0.7 |
|  | United Kingdom | 90 | 37.8 | 123 | 40.5 |
|  | United States | 106 | 44.5 | 117 | 38.5 |
|  | Zimbabwe | 0 | 0.0 | 2 | 0.7 |

Comparison of medians and distribution of scores of those who did and did not complete Round 2

| **Outcome** | **Median score of those who**  **did not complete Round 2** | **Median score of those who**  **completed Round 2** | **Mann-Whitney U (Test for Median)** | **Did not complete Round 2,**  **% of Participants scoring 7-9** | **Completed Round 2,**  **% of Participants scoring 7-9** |
| --- | --- | --- | --- | --- | --- |
| **Labour and birth outcomes** | | |  |  |  |
| Induction of birth | 7.00 | 6.00 | .004 | 54.2% | 40.7% |
| Length of time from identification of a stillbirth to the birth | 7.00 | 7.00 | .129 | 59.8% | 52.7% |
| Pain relief for labour and birth | 7.00 | 7.00 | .013 | 58.3% | 50.2% |
| Type of birth | 7.00 | 7.00 | .098 | 60.2% | 52.9% |
| Complications during birth for mother or baby | 8.00 | 8.00 | .445 | 74.0% | 71.3% |
| **Postpartum maternal medical outcomes** | | |  |  |  |
| Maternal complications after birth | 8.00 | 7.00 | .087 | 68.3% | 66.2% |
| Maternal life-threatening complications after birth | 8.00 | 8.00 | .846 | 78.8% | 79.4% |
| Length of maternal hospital stay due to medical complications after a stillbirth | 6.00 | 6.00 | .367 | 46.6% | 42.2% |
| Maternal postpartum recovery | 7.00 | 7.00 | .095 | 64.0% | 61.1% |
| Maternal long term physical complications related to the birth | 7.00 | 7.00 | .610 | 61.3% | 56.0% |
| Maternal long term physical health outcomes | 7.00 | 7.00 | .427 | 62.8% | 52.9% |
| Maternal death | 8.00 | 8.00 | .804 | 85.2% | 82.6% |
| **Care experience outcomes** | | |  |  |  |
| Parents' experience of their care following stillbirth | 8.00 | 8.00 | .817 | 88.3% | 88.6% |
| Parents' experience of communication with care professionals | 8.00 | 8.00 | .212 | 86.4% | 91.3% |
| Parents' experience of support from care professionals | 9.00 | 8.00 | ^.a^ | 88.8% | 92.6% |
| Perceived acknowledgement of parenthood by care professionals | 8.00 | 8.00 | .257 | 79.5% | 78.5% |
| Perceived acknowledgment of baby by care professionals | 9.00 | 8.00 | ^.a^ | 84.9% | 84.8% |
| Impact of providing stillbirth care on healthcare professionals' | 8.00 | 8.00 | .127 | 77.7% | 72.0% |
| Formal complaints or legal action | 7.00 | 6.00 | .251 | 58.1% | 49.7% |
| **Investigation outcomes** | | |  |  |  |
| Uptake of medical investigations performed to understand why a baby died | 9.00 | 8.00 | .008 | 87.9% | 79.7% |
| Hospital review carried out by healthcare professionals to help understand why the baby died | 9.00 | 8.00 | .027 | 90.1% | 81.7% |
| Uptake of parental engagement in the hospital review to understand why a baby died | 8.00 | 8.00 | .097 | 80.7% | 70.3% |
| Cause of death identified | 9.00 | 8.00 | ^.a^ | 89.4% | 84.8% |
| Parents' understanding of why their baby died | 9.00 | 8.00 | <001 | 88.9% | 85.1% |
| Parents' understanding about stillbirth | 9.00 | 8.00 | .002 | 88.4% | 81.4% |
| Improvements to care and patient safety | 9.00 | 9.00 | ^.a^ | 92.6% | 86.4% |
| **Grief outcomes** | | |  |  |  |
| Overwhelming or complicated grief | 8.00 | 8.00 | .031 | 82.7% | 79.2% |
| Coping with grief | 8.00 | 8.00 | .401 | 85.9% | 76.0% |
| Feelings of self-blame, guilt or failure | 8.00 | 8.00 | .057 | 82.3% | 76.5% |
| Perceived acknowledgment of grief by others | 8.00 | 8.00 | .165 | 75.1% | 66.9% |
| Grief of whole family | 8.00 | 7.00 | .227 | 70.8% | 61.1% |
| **Mental health outcomes** | | |  |  |  |
| Depression | 8.00 | 8.00 | .024 | 78.9% | 75.3% |
| Anxiety | 8.00 | 8.00 | .016 | 79.4% | 74.4% |
| Post-traumatic stress disorder | 8.00 | 8.00 | .016 | 85.0% | 80.4% |
| Suicidal thoughts, attempted suicide, suicide | 9.00 | 8.00 | .070 | 86.5% | 80.7% |
| Other mental health difficulties | 8.00 | 7.00 | .026 | 76.0% | 69.6% |
| Drug and alcohol use | 7.00 | 7.00 | .208 | 70.1% | 61.9% |
| Mental functioning | 8.00 | 7.00 | .155 | 71.7% | 64.8% |
| Any mental health treatment (including type) | 8.00 | 7.00 | .014 | 76.0% | 66.4% |
| **Emotional outcomes** | | |  |  |  |
| Emotional wellbeing | 8.00 | 7.00 | .010 | 70.1% | 64.0% |
| Self esteem | 7.00 | 7.00 | .040 | 62.1% | 55.0% |
| Body confidence | 6.00 | 6.00 | .329 | 44.1% | 39.0% |
| Sense of control | 7.00 | 7.00 | .018 | 61.0% | 50.9% |
| Stress | 7.00 | 7.00 | .031 | 68.4% | 60.6% |
| Sexual wellbeing | 6.00 | 6.00 | .038 | 44.6% | 34.5% |
| **Whole person outcomes** | | |  |  |  |
| Impact on identity | 7.00 | 6.00 | .300 | 58.0% | 49.5% |
| Adjustment to new normal | 7.00 | 7.00 | .190 | 64.4% | 58.8% |
| Personal growth or positive impact | 6.50 | 6.00 | .068 | 50.0% | 40.8% |
| Impact on spirituality | 6.00 | 5.00 | .049 | 35.1% | 31.1% |
| Physical wellbeing | 7.00 | 7.00 | .116 | 60.9% | 53.4% |
| Quality of life | 7.00 | 7.00 | .418 | 65.5% | 60.8% |
| **Social outcomes** | | |  |  |  |
| Social impact | 7.00 | 7.00 | .334 | 54.7% | 54.2% |
| Opportunities to talk about stillbirth experience with others | 8.00 | 7.00 | .082 | 79.5% | 70.6% |
| Degree of isolation | 7.00 | 7.00 | .103 | 70.2% | 67.4% |
| Perceived stigma from community | 7.00 | 7.00 | .106 | 67.1% | 59.6% |
| Impact on work | 7.00 | 7.00 | .129 | 67.3% | 58.4% |
| **Relationship and support outcomes** | | |  |  |  |
| Impact on relationship with partner | 7.00 | 7.00 | .568 | 65.5% | 69.8% |
| Perceived support from partner | 7.00 | 7.00 | .915 | 63.4% | 62.6% |
| Impact on relationships with family, friends and community | 7.00 | 7.00 | .140 | 60.6% | 50.9% |
| Perceived support from family, friends and community | 7.00 | 6.00 | .362 | 60.2% | 49.5% |
| Perceived support with returning to work | 7.00 | 7.00 | .818 | 64.6% | 55.0% |
| Satisfaction with support resources and support groups | 7.00 | 7.00 | .787 | 59.4% | 57.3% |
| **Older children outcomes** | | |  |  |  |
| Psychological health of older children in the family | 7.00 | 7.00 | .666 | 67.0% | 62.4% |
| Impact on parenting | 7.00 | 7.00 | .595 | 70.1% | 64.8% |
| Support for older children and parenting | 8.00 | 7.00 | .366 | 68.4% | 56.4% |
| **Economic outcomes** | | |  |  |  |
| Financial costs for parents | 7.00 | 7.00 | .394 | 66.0% | 50.2% |
| Financial costs for health service and wider society | 6.00 | 6.00 | .026 | 49.1% | 37.7% |
| **Twin or multiple outcomes** | | |  |  |  |
| Survival of baby/ies after stillbirth is identified in a multiple pregnancy | 7.00 | 8.00 | .344 | 69.4% | 74.2% |
| Preterm birth of surviving baby(ies) after stillbirth is identified in a multiple pregnancy | 7.00 | 7.00 | .794 | 69.4% | 67.7% |
| Pregnancy complications that risk the life of the surviving baby(ies) after stillbirth is identified in a multiple pregnancy | 8.00 | 8.00 | .342 | 72.9% | 75.6% |
| Pregnancy complications for the mother after stillbirth is identified in a multiple pregnancy | 8.00 | 8.00 | .841 | 71.4% | 74.6% |
| Neonatal outcomes of surviving baby(ies) after stillbirth is identified in a multiple pregnancy | 8.00 | 8.00 | .892 | 67.3% | 77.7% |
| Neurodevelopment of surviving baby(ies) after stillbirth is identified in a multiple pregnancy | 7.00 | 7.00 | .860 | 61.2% | 65.9% |
| Medical health of surviving baby(ies) after stillbirth is identified in a multiple pregnancy | 7.00 | 7.00 | .797 | 61.2% | 66.9% |
| Attachment to surviving baby(ies) after stillbirth is identified in a multiple pregnancy | 7.00 | 7.00 | .742 | 64.6% | 60.6% |
| Psychological health of surviving child after stillbirth is identified in a multiple pregnancy | 7.00 | 7.00 | .942 | 53.1% | 52.3% |
| **Planning subsequent pregnancy outcomes** | | |  |  |  |
| Perceived support for planning next pregnancy after stillbirth | 8.00 | 8.00 | .183 | 84.0% | 76.7% |
| Need for fertility treatment after stillbirth | 7.00 | 6.00 | .424 | 57.9% | 49.6% |
| Infertility | 7.00 | 6.00 | .069 | 63.3% | 49.1% |
| Parents choosing not to become pregnant again after a stillbirth | 7.00 | 6.00 | .058 | 54.3% | 44.5% |
| **Subsequent pregnancy outcomes** | | |  |  |  |
| Time between stillbirth and next pregnancy | 6.00 | 6.00 | .996 | 47.1% | 47.9% |
| Number of pregnancies between stillbirth and live birth | 6.00 | 6.50 | .959 | 48.8% | 50.0% |
| Complications for the baby in a subsequent pregnancy after stillbirth | 8.00 | 8.00 | .954 | 81.0% | 82.4% |
| Complications for the mother in a subsequent pregnancy after stillbirth | 8.00 | 8.00 | .909 | 82.4% | 79.8% |
| Preterm birth in a subsequent pregnancy after stillbirth | 7.50 | 7.00 | .697 | 69.0% | 71.2% |
| Induction of labour in a subsequent pregnancy after stillbirth | 7.00 | 7.00 | .678 | 58.8% | 54.9% |
| Type of birth in a subsequent pregnancy after stillbirth | 7.00 | 6.00 | .068 | 60.0% | 49.7% |
| Birth and postpartum complications in a subsequent pregnancy after stillbirth | 7.00 | 7.00 | .879 | 62.4% | 62.2% |
| Survival of baby in a subsequent pregnancy after stillbirth | 8.00 | 8.00 | .551 | 81.2% | 84.5% |
| Newborn outcomes in baby born after stillbirth | 8.00 | 8.00 | .358 | 71.8% | 71.9% |
| Additional scans and clinic appointments during subsequent pregnancy after stillbirth | 8.00 | 8.00 | .084 | 80.0% | 70.5% |
| Unplanned hospital admission prior to birth of baby in a subsequent pregnancy after stillbirth | 7.00 | 7.00 | .360 | 69.4% | 59.3% |
| Perceived support for subsequent pregnancy, birth and parenthood after stillbirth | 8.00 | 7.00 | .061 | 69.4% | 63.4% |
| Coping in a subsequent pregnancy after stillbirth | 8.00 | 8.00 | .055 | 81.2% | 77.8% |
| Attachment to baby during subsequent pregnancy after stillbirth | 8.00 | 8.00 | .027 | 69.0% | 68.9% |
| Parents' satisfaction with care in a subsequent pregnancy after stillbirth | 8.00 | 7.00 | .075 | 70.2% | 68.2% |
| **Subsequent children outcomes** | | |  |  |  |
| Development of child born after a stillbirth | 7.00 | 5.00 | .007 | 51.5% | 34.6% |
| Medical health of child born after stillbirth | 7.00 | 6.00 | .019 | 52.0% | 37.1% |
| Attachment to a child born after stillbirth | 7.00 | 7.00 | .284 | 63.0% | 54.9% |
| Psychological health of child born after stillbirth | 7.00 | 6.00 | .135 | 55.0% | 45.1% |

^a – All test field values are less than or equal to the median^
