# Supplementary material for: A Core Outcome Set for Stillbirth Care: An International Consensus Study
Source: BJOG. 2025 Jul 7;132(13):2149–59. doi: 10.1111/1471-0528.18265 (PMC12592755; doi:10.1111/1471-0528.18265)
Supplement: Supplementary file 6 — Appendix S6. [file BJO-132-2149-s007.docx]

|  | Country of attendance | Number of parents or family members | Number of professionals |
| --- | --- | --- | --- |
| 1^st^ Consensus meeting (Parents only) | | | |
|  | United Kingdom | 4 | 0 |
|  | United States | 8 | 0 |
| 2^nd^ Consensus meeting (Parents and professionals) | | | |
|  | Australia | 2 | 1 |
|  | Ghana | 0 | 1 |
|  | United Kingdom | 12 | 7 |
|  | United States | 6 | 2 |
| 3^rd^ Consensus meeting subsequent pregnancy care (Parents and professionals) | | | |
|  | Australia | 1 | 1 |
|  | Brazil | 0 | 1 |
|  | Ghana | 0 | 2 |
|  | Republic of Ireland | 0 | 2 |
|  | India | 0 | 1 |
|  | Russia | 0 | 1 |
|  | United Kingdom | 3 | 5 |
|  | United States | 2 | 0 |
| 4^th^ Consensus meeting when a stillbirth occurs in a multiple pregnancy(Parents and professionals) | | | |
|  | Australia | 1 | 1 |
|  | Brazil | 0 | 0 |
|  | Italy | 0 | 1 |
|  | Republic of Ireland | 0 | 1 |
|  | India | 0 | 0 |
|  | Russia | 0 | 0 |
|  | United Kingdom | 3 | 5 |
|  | United States | 1 | 0 |
