# Supplementary material for: A Core Outcome Set for Stillbirth Care: An International Consensus Study
Source: BJOG. 2025 Jul 7;132(13):2149–59. doi: 10.1111/1471-0528.18265 (PMC12592755; doi:10.1111/1471-0528.18265)
Supplement: Supplementary file 7 — Appendix S7. [file BJO-132-2149-s006.docx]

Results from 1^st^ Consensus Meeting – Parents only

| **Outcomes Voted On** | **Total No. of Voters** | **Consensus out** | **No consensus** | **Consensus in** | **Notes & Quotes** |
| --- | --- | --- | --- | --- | --- |
| Medical complications for mother and baby | 15 | 0 | 4 (27%) | 11 (73%) | - Parents agreed that medical complications for mother should be combined with maternal death |
| Maternal Death (Separate Outcome) | 15 | 2 (13%) | 9 (60%) | 4 (27%) | - Vote conducted on maternal death as separate outcome |
| Parents’ experience of care and support | 15 | 0 | 2 (13%) | 13 (87%) | - Parents wanted two outcomes ‘Parent’s experience of care and support’ and ‘Parent’s experience of communication and shared decision making’ |
| Parents’ experience of communication and shared decision making | 15 | 0 | 1 (7%) | 14 (93%) |  |
| Perceived acknowledgement of parenthood & baby | 15 | 1 (7%) | 0 | 14 (93%) | - Agreed outcomes could be combined - Parent, USA *“Absolutely if you acknowledge the baby as a person that does intrinsically acknowledge your parenthood, but I think a lot of times if you don’t have another living baby at home your parenthood is really invisible to the world. So, I think from my own anecdotal experience they both feel very important to me.”* |
| Impact of providing stillbirth care on healthcare professionals | 14 | 0 | 7 (50%) | 7 (50%) | - Mixed opinions on outcome - Parent, USA *“The better care they get the better care they give.”* - Parent, USA *“Her doctor said, ‘these things happen’, dismissed it.”* |
| Trust in health care professionals | 14 | 1 (7%) | 10 (71%) | 3 (21%) | - Parents felt difficult one to measure as interlinked with care experience - Trust varies and related to timing in relation to stillbirth - Parent, UK *“After losing [name] my trust in the healthcare profession plummeted, so minus a million. But then working with a much better healthcare professional it went back up, and as I said before it’s case by case and it’s all time dependent as well. So, I am trying to picture what research… how this would help a research project, what question would you be asking?”* |
| Dimensions of grief | 14 | 0 | 0 | 14 (100%) | - Discussion on whether outcomes of grief should be combined - ‘Self-blame, guilt and failure’ raised a ‘unique’ aspect of grieving in stillbirth - Parent, USA *“Because everybody does grieve so individually including combining them is probably an okay thing, because not everybody is going to have the same specific elements or reactions, but it’s still grief, and so if somebody studying grief specifically then they would probably want to tease out some more of those things, but if it’s a more general very high-level type thing I think understanding all the different variables that can go into grief can be seen as fairly equal.”* - Voted unanimously to combine specific grief outcomes |
| Mental Health | 14 | 0 | 2 (14%) | 12 (86%) | - Agreement mental health outcomes should be combined |
| Mental health and emotional wellbeing | 14 | 0 | 1 (7%) | 13 (93%) | - Agreement mental health and emotional wellbeing should be combined - One parent felt mental and emotional wellbeing were different |
| Perceived opportunities to talk about stillbirth experience with others | 11 | 0 | 2 (18%) | 9 (82%) | - Important outcome related to ‘ending stigma’ and ‘taboo’ around stillbirth - Parents unsure about wording of ‘Perceived opportunities to talk about stillbirth experience’ - Parent, USA *“I would never say the stillbirth experience, I would say the death and birth of my daughter.”* - Parent, USA *“The most important thing to talk about it’s the societal acceptance of your child as another human being in the community who died that’s the primary important thing.”* - One suggestion *“perceived opportunities to help break the silence and stigma surrounding stillbirth”* - To discuss further in next meeting and reword ‘stillbirth experience’ |
| Degree of isolation | 15 | 0 | 11 (73%) | 4 (27%) | - Some parents felt isolation was interlinked with mental health, grief, taboo and recognition of parenthood and opportunity to talk about stillbirth experience |
| Perceived opportunities to talk about stillbirth and isolation | 15 | 0 | 2 (13%) | 13 (87%) | - Voted for isolation to be potentially a combined outcome with opportunities to talk about stillbirth - To consider wording for next consensus meeting |
| Impact on work | 12 | 2 (17%) | 8 (67%) | 2 (17%) | - Parents unsure initially if should be separate outcome and should be under ‘social functioning’ |
| Impact on work (re-vote) | 12 | 0 | 4 (33%) | 8 (67%) | - Re-vote took place as some parents felt that it was important to be distinct to raise awareness of the impact on work and could lead to more support/intervention from government - Parent, UK: *“If research shows that actually parents struggle to go back to work, or they change jobs, or they lower incomes or whatever after experiencing the loss of a baby then there might be more likely to be support for families who have lost a baby, and there might be more research into how to reduce stillbirth …. in a way that some of the other questions won’t ever actually necessarily generate new interventions or support from government. Because people tend to be quite obsessed with work and employment and whether or not people can go to work, earn money and contribute to the capitalist system.”* |
| Impact on relationship and perceived support from partner | 12 | 0 | 6 (50%) | 6 (50%) | - Some parents stated it was not applicable in their situation raising the question as should it be measured in all studies |
| Impact on children and parenting | 8 | 1 (13%) | 4 (50%) | 3 (38%) | - Some parents stated it was not applicable in their situation and queried whether it could be grouped with above |
| Impact on relationship with immediate family (close partner, family members and children) | 11 | 0 | 3 (27%) | 8 (73%) | - Parent, US: *“I feel like the impact on the family unit as a whole, the relationship of the parents, the relationship of the older siblings to the dead child, even the grandparents, maybe is that what we’re trying to get at, the health of the family unit? I think potentially you could have the health of the relationship and the mental health of the siblings under that.”* |
| Perceived support for planning next pregnancy after stillbirth | 11 | 0 | 5 (45%) | 6 (55%) | - Parents felt this could be grouped under broader category of care experience and support |
| Uptake of investigations performed to understand why a baby died | 11 | 0 | 3 (27%) | 8 (73%) | - Agreed uptake of medical investigations, hospital review and parental engagement should be combined |
| Cause of death identified | 11 | 0 | 1 (9%) | 10 (91%) | - Parent, USA: *“I just think it’s so unacceptable that so many of these deaths are just termed unexplained, shrug emoji, explain it. I think that we need to shout from the rooftops that they should not be unexplained so that they don’t happen to other families in the future, and other babies.”* |
| Parents’ understanding about the cause of death | 11 | 0 | 1 (9%) | 10 (91%) | - Agree should be core outcome |
| Additional counselling or subsequent pregnancy care advice following investigations | 11 | 0 | 9 (82%) | 2 (18%) | - Parents felt this outcome was covered in the ‘Parents’ understanding about the cause of death’ |
| Length of time investigations into understanding why a baby died continue after a stillbirth | 11 | 2 (18%) | 8 (73%) | 1 (9%) | - Some parents were unsure if a short length of time is a good or bad thing - Parent, USA: *“I think does this have something to do with the investigations like one day long, and then they come back and they say it’s unexplained, or… what is it measuring, is it saying it’s taking too long to do it because nobody is prioritising it, or is it saying it’s way too short to do it because they are just not doing anything.”* |
| Improvements to care and patient safety | 11 | 0 | 2 (18%) | 9 (82%) | - Agreed should be core outcome |

Results from 2^nd^ Consensus meeting – Parents and Professionals

| **Outcomes Voted On** | **Total No. of Voters** | **Consensus out** | **No consensus** | **Consensus in** | **Notes & Quotes** |
| --- | --- | --- | --- | --- | --- |
| Medical complications for mother and baby | 28 | 0 | 10 (36%) | 18 (64%) | - Some debate on relevance of outcome in different settings - Advised to remove ‘baby’ from the outcome - Parent, UK: *“I think basically maternal death and medical complications for the mother are personally my most important outcome during the experience, so I think you probably should include them, I don’t know, from a male perspective the only thing I really wanted was my partner to get through it alright and in one piece as my primary concern. So it’s a nice hard endpoint as well.”* |
| Medical complications just for mother | 28 | 0 | 11 (39%) | 17 (61%) | - Obstetrician: *“All I was going to say was probably really echo what you said [chair], if we think about this as a core outcome set I would see it as looking at this would be management of birth and the intervention might be a better way of induction, and then we would be looking at hopefully less maternal complications. So that’s how I see this being important in the core outcome set.”* |
| Maternal death | 28 | 1 (4%) | 13 (46%) | 14 (50%) | - Voted consensus out as separate outcome |
| Life-threatening complications and maternal death | 27 | 1 (4%) | 2 (7%) | 24 (89%) | - Discussion as no physiological/medical outcome in core outcome set - Stakeholders suggested new outcome ‘Life-threatening complications and maternal death’ - Parent, UK: *“It concerns me that I know we’re concerned about what you might call the softer outcomes, so effect to family and psychological outcomes, but at the end of the day if a mother is not alive after a stillbirth then we really need to be looking at the care she is receiving, or if she’s exsanguinated to the extent she needs to go to ITU then we need to be thinking about how she was cared for. So I think this is the first and foremost outcome to me.”* |
| Non-life threatening maternal medical complications | 26 | 4 (15%) | 14 (54%) | 8 (31%) | - Voted consensus out |
| Parent’s experience of respectful care and support | 26 | 0 (0%) | 1. (19%) | 21 (81%) | - Discussed combining parents' experience of communication with care professionals, parents' experience of support from care professionals, parents' experience of shared decision making, perceived acknowledgment of parenthood and baby - Discussed the outcome ‘Parents’ experience of respectful care and support’ and framework developed by Boyle et al (2020)^20^. - Discussion on whether the outcome is feasible to measure as it encompasses a range of outcomes - Professional: *“I think this is such an important outcome and it does cover quite a lot but I think if we were to separate them out it would be really difficult to just pick a few or pick one that was the most important to go forward. I appreciate it’s probably a difficult thing to measure in practice when it’s all together like this in one way, but I am not sure that any one of these elements potentially are able to say it’s more important than another.”* - Important each outcome is described in the definition including explicitly recognition of parenthood and baby |
| Trust in health care professionals | 25 | 0 (0%) | 17 (68%) | 8 (32%) | - Vote on trust as separate outcome - Parent, USA: *“I do remember in the parent meeting I believe we were talking about how trust is tricky because to be honest in any other circumstances I could trust the doctor, but after I lose my child I don’t trust any doctor no matter how good or amazing they are, I have just lost that ability to trust the medical professionals.”* - Discussion on how it could be combined with parents’ experience of respectful care and support |
| Grief | 25 | 0 (0%) | 3 (12%) | 22 (88%) | - Some stakeholders did not like the ‘word dimensions’, preferred ‘grief’ on its own - Agreement specific outcomes of grief should be combined - Psychologist, UK: *“The dimensions always conjures up more of this and less of that, and I don’t think that’s what’s it’s about.”* - Parent, Aus: *“Being a grief counsellor for many years I think this is the way to go to bring all of those things together. There are good tools for measuring grief, you have put individuals and whole families, and I think this is really critical.”* |
| Mental health and emotional wellbeing | 25 | 0 (0%) | 1 (4%) | 24 (96%) | - Agreement by stakeholders to combine mental health and emotional wellbeing outcomes - Psychologist: *“I think that phrasing works perfectly well, and then people can measure the different… and look at the different aspects, because studies will be different. Because the symptoms are different at different points in time, different trajectories, different patterns for different individuals, I think this general term is the right one, that’s what I would argue for.”* |
| Degree of isolation | 26 | 0 (0%) | 8 (31%) | 18 (69%) | - Stakeholders did not like the word ‘degree’ in isolation |
| Perceived opportunities to talk about stillbirth experience with others | 25 | 0 (0%) | 7 (28%) | 18 (72%) | - Midwife: *“[Re: opportunities to talk about stillbirth] These things are in the stillbirth stigma scale, so if you have the stillbirth stigma scale as the measure then you capture all this.”* - Would be potentially difficult to measure |
| Isolation & Stigma | 24 | 1 (4%) | 3 (13%) | 21 (88%) | - Discussion on whether isolation is a symptom of stigma or whether it is separate from each other - Midwife: *“I feel very much that this is more than isolation, that it’s actually a symptom of stigma, and that parents don’t feel like they can relate to others because others can’t relate to them, they feel self-blame, they feel devalued, they feel like they can’t disclose that they have had a stillbirth or that if they do disclose that people don’t want to listen to that story, and that’s all part of stigma.”* - Stigma originally consensus out but only marginally (74.3% professionals scored critical) - Stakeholders felt ‘Stigma’ more encompassing outcome – as could include opportunities to talk about stillbirth - Parent, USA: *“[Re: opportunities to talk about stillbirth] I think as far as ending the stigma around stillbirth is important, and also saving future babies]* - Vote conducted on ‘Isolation and Stigma’ |
| Impact on work | 25 | 1 (4%) | 4 (16%) | 20 (80%) | - Discussed differing of opinion between professionals and parents - Relevance to low resource settings, work may not be paid work, could be “*work in the house, on the farm, in the fields”* - Midwife, Ghana: *“Yeah, in Ghana when someone has stillbirth that person unfortunately is not allowed to grieve for a long time, so they try to go back to their work, and that’s why I would think that to be critical to how this affects them.”* - Discussed feasibility as long-term outcome - Midwife: “*I guess I was fairly interested in seeing this result because if you’re a health professional without the experience of bereavement you can only imagine what the impact on job, unemployment, change of career, workplace discrimination and all that sort of stuff has on you, so it’s not at all surprising to me to see that parents rank this much more highly than the professionals, because unless you’re a social worker or something you wouldn’t necessarily know about all of these things that happen months/years, and decades after you have lost your baby.”* |
| Impact on relationship and perceived support from partner | 25 | 0 (0%) | 9 (36%) | 16 (64%) | - Debate on whether impact on relationship and support from partner should be included as separate or with impact on immediate family - Psychologist: *“The partner stuff needs to be separate really. When we ask parents about where they got most of their support from their partner was the key.”* |
| Impact on children and parenting | 21 | 0 (0%) | 9 (43%) | 12 (57%) | - Debate on whether impact on children and parenting should be separate or with impact on immediate family - Parent, USA: *“We decided that because families take so many different forms it made more sense to look at family support and how families are supported, be they children, which is critically important, I say as somebody without any other children except for my one daughter who died, be they partners which is critically important, I would say that as a single mum.”* |
| Impact on relationship with immediate family: for example, partner, children, siblings, grandparents, close friends | 22 | 0 | 4 (18%) | 18 (82%) | - Agreement by stakeholder’s outcome should be very “broad and flexible” |
| Uptake of investigations performed to understand why a baby died | 18 | 2 (11%) | 9 (50%) | 7 (39%) | - Discussion on how investigation outcomes are specific and would not be specific in all circumstances, for example counselling or subsequent pregnancy - Discussed framework of outcomes and onion – investigation outcomes could be mandatory in specific circumstances. Agreement by stakeholder’s good idea to apply framework - Discussed different international contexts – some countries parents have to pay for investigations so may not be a good measurement of care/intervention |
| Cause of death identified | 20 | 0 (0%) | 3 (15%) | 17 (85%) | - Discussion around some stillbirths may be ‘unexplained’ despite investigations - Parent, USA: *“We want our babies to be treated like human beings, like any other human being, and so if a human being died we want to know why that human being died, and that’s definitely what happens when someone dies in a hospital setting, and I think that’s what we’re trying to say. So the question of what happens when an adult dies, like you have to find the cause, we just want our child to be treated like a person, and we don’t want our child to be treated like an unfortunate blip that happened to the parent.”* |
| Parents’ understanding about the cause of death | 20 | (0%) | 3 (15%) | 17 (85%) | - Parent, USA *“I also think that parents’ understanding is really crucial to parents’ wellbeing.”* |
| Impact of providing stillbirth care on healthcare professionals | 23 | 1 (4%) | 17 (74%) | 5 (22%) | - Agreement not ‘core’ outcome by stakeholders |
| Length of time investigations into understanding why a baby died continue after a stillbirth | 18 | 3 (17%) | 15 (83%) | 0 (0%) | - Debate on length of time whether positive or negative outcome - Psychologist*: “The thing is that the length of time matters enormously to parents when they are going through it and the delays.”* - Obstetrician: *“I think it matters a lot at the time, but if you have got an answer in two weeks and then found out it was sloppy and meaningless you would then worry about where that happened.”* |
| Improvements to care and patient safety | 20 | 1 (5%) | 6 (30%) | 13 (65%) | - Agreement important, however difficult to measure as too broad - Debate between parents and professionals about whether an outcome or not - Not really an outcome more of *a “conclusion”* in a research study - Discussion on whether it should be an aspirational outcome |

Results of 3^rd^ Consensus meeting – Outcomes for subsequent pregnancy care after stillbirth

| **Outcomes Voted On (Subsequent Pregnancy)** | **Total No. of Voters** | **Consensus out** | **No Consensus** | **Consensus in** | **Notes & Quotes** |
| --- | --- | --- | --- | --- | --- |
| General discussion on outcome framework  for subsequent pregnancy addition | n/a | n/a | n/a | n/a | - Clarification on how outcome framework/onion could work - Midwife: *“I don’t think we can talk about stillbirth and pregnancy loss without considering subsequent pregnancy... because they are so interlinked, and women and couples will… the majority of whom will proceed to a subsequent pregnancy very soon after their index loss. So the index loss obviously is the lens through which subsequent decisions are made”* - Agreement amongst stakeholders that subsequent pregnancy care outcomes should be “embedded” in core outcome set but consideration needs to be given on how “final product should be presented” |
| Complications for the baby in a subsequent pregnancy after stillbirth | 18 | 0 (0%) | 1 (6%) | 17 (94%) | - Discussion on how outcome broad - Definition would need refining to ensure similar outcomes are measured - Parent, USA: *“Critical in understanding full picture”* - Researcher: *“I see this as a critically important outcome, and a baby in a subsequent pregnancy after stillbirth is at higher risk of all sorts of complications, and I think that’s really important to capture, so I just wanted to add that.”* |
| Complications for the mother in a subsequent pregnancy after stillbirth | 18 | 0 (0%) | 2 (11%) | 16 (89%) | - Discussion on how outcome broad - Definition would need refining to ensure similar outcomes are measured |
| Preterm birth in a subsequent pregnancy after stillbirth | 18 | 3 (17%) | 6 (33%) | 9 (50%) | - Discussed whether outcome could be included in with ‘Complications for the mother in a subsequent pregnancy after stillbirth’ or whether gestational age should be measured as a non-binary outcome - Parent, UK: *“I guess I wondered what the rational for making this a binary outcome? I know that’s a definition of preterm, but would it not be more valuable to actually just collect the gestational age that the baby was born at? And then I guess also on the thing around complications, presumably some of those are potentially related to that gestational age. I know it’s anecdotal but all of my three subsequent pregnancies were induced between 36 and 37 weeks, and they all had complications, but that was probably because of that gestational age rather than anything else, although hard to know.”* - Discussion on the different medical and psychological reasons for pre-term birth - Discussion on whether gestational age is an outcome |
| Vote on preterm birth as composite outcome for complications for mother | 18 | 1 (6%) | 7 (44%) | 10 (56%) | - Vote on whether preterm birth should be composite outcome in complications for mother/baby |
| Vote on gestational age as separate outcome | 17 | 1 (6%) | 7(41%) | 9 (53%) | - Vote on whether gestational age should be a separate outcome - Parent, USA: “It’s illegal to induce before 39 weeks without a medical reason in her state.” |
| Birth and postpartum complications in a subsequent pregnancy after stillbirth | 18 | 3 (17%) | 7 (39%) | 8 (44%) | - Discussed importance of not just physiological but psychological outcomes as well - Parent, Aus: *“Yeah, I think in all of these outcomes it’s like birth is not just a physiological thing that occurs to a woman, it’s a transformative, it’s there’s so much at play here, and I think balanced outcomes which doesn’t just consider the physiological.”* |
| Survival of baby in a subsequent pregnancy after stillbirth | 18 | 0 (0%) | 2 (11%) | 16 (89%) | - Agreement important outcome by stakeholders - Voted as core outcome |
| Newborn (neonatal) outcomes in baby born after stillbirth | 18 | 0 (0%) | 4 (22%) | 14 (78%) | - Midwife: *“So I see the need of these outcomes for the fact that could help in the identification of near misses, is as we know is mums that have had a… pregnant people that have had a stillbirth are at higher risk of having another one.”* - Discussed need for resuscitation in definition of outcome |
| Additional scans and clinic appointments during subsequent pregnancy after stillbirth | 17 | 2 (12%) | 6 (35%) | 9 (53%) | - Midwife: “*Just to say we have lots of, well not lots but we have lots of empirical evidence to say that this is a very important outcome to measure, that resource… pregnancy after loss has major resource implications for healthcare services, because women and pregnant people themselves require additional reassurances, because this is normal.”* - Agreement important outcome however uncertain if should be ‘core’ outcome as additional scans are very personalised to individuals’ care and also can depend on jurisdiction. - Parent, Aus: *“I was just thinking of this as like an outcome though in research, and for researchers. I don’t 100% see the value, and I myself had many additional scans and saw both the side of the reassurance and then 20 minutes later saw the fact that I wanted to just live with an ultrasound in my hand really... so one person may have needed 20 scans, and another person have only needed the average two, that might not have indicated any difference of health and baby but maybe emotional health and reassurance.”* - Midwife: *“What is normal across different jurisdictions in relation to number of scans, and I suppose just a final point, and I should have asked this at the beginning, and I didn’t, but I am assuming this is a core outcome set that we are going to suggest or you as the research team are going to suggest is applicable across all settings, low, high, middle income countries, and we just need to be also mindful of that if we’re setting core outcomes.”* |
| Unplanned hospital admission prior to birth of baby in a subsequent pregnancy after stillbirth | 17 | 2 (12%) | 12 (71%) | 3 (18%) | - Professional: *“It is absolutely empowering them, I totally agree, it is empowering them, but also that they feel empowered to also be able to go to the hospitals if they are unsure, or if they need that reassurance.”* |
| Perceived support for subsequent pregnancy, birth and parenthood after stillbirth  Parents' satisfaction with care in a subsequent pregnancy after stillbirth | n/a | n/a | n/a | n/a | - Discussion on whether outcome could be combined with ‘Parents’ experience of respectful and supportive care’ - Stakeholders in agreement outcomes already covered - To ensure outcome covered in definition - Vote on whether outcome covered: 16 (94%) Yes, 1 (6%) No |
| Coping in a subsequent pregnancy after stillbirth  Anxiety related to subsequent pregnancy and children | n/a | n/a | n/a | n/a | - Discussion on whether outcome covered in ‘Mental health and emotional wellbeing’ outcome - Parent, USA: *“Says if we’re saying these are already covered in the core outcomes we should have a language that specifically includes it in relation to pregnancy after loss.”* - To ensure mental health is covered in after subsequent birth as well - Vote on whether outcome covered in ‘Mental health and emotional wellbeing’: 100% (17) Yes |
| Attachment to baby during subsequent pregnancy after stillbirth | 16 | 0 (0%) | 4 (25%) | 12 (75%) | - Discussion on importance during subsequent pregnancy and after birth - Parent, Aus: “*I think this is incredibly important and not very well looked at. I think it’s the old adage of once you have a live baby in your hands everything is meant to be well and good but we know that’s not always the case. So I think this helps by adding it to the core outcomes it highlights the issue that this needs to be looked at further, yeah I think it’s critical.”* |

Results of 4^th^ Consensus meeting – Outcomes for when a stillbirth occurs in a multiple pregnancy

| **Outcomes Voted On** | **Total No. of Voters** | **Consensus out** | **No Consensus** | **Consensus in** | **Notes & Quotes** |
| --- | --- | --- | --- | --- | --- |
| Survival of baby/ies | 13 | 0% | 1 (8%) | 12 (92%) | - Discussion on definition to include live birth, miscarriage, stillbirth and neonatal death - Researcher: *“It keeps being mentioned in a number of projects I’ve been involved, which is the concept of one outcome as you said which is take home healthy live term baby, which means take out all the morbidity, extreme prematurity, the risk of stillbirth, and this is really what women are interested in.”* |
| Preterm birth | 13 | 0% | 1 (8%) | 12 (92%) | - Researcher*: “I was just thinking it might be worth collecting more information about earlier preterm birth, because in a multiple pregnancy so many pregnancies will end before 37 weeks anyway I would have thought, 37/38, that actually you’d have a really high proportion of multiples being preterm anyway, that maybe having a bit gradation on the gestation, so maybe just noting whether they were very preterm or extremely preterm might be more helpful?”* - Discussion on whether to combine preterm birth and survival – important to measure separately including nuance around it - Researcher: *“The impact of a loss on the other twin is vital to capture, and prematurity would be a significant morbidity that we see with those surviving twins, and sometimes they get born earlier because of that. So I really think prematurity is vital to capture for the health of the other twin, as well as the management of the pregnancy.”* |
| Pregnancy complications that risk the life of the surviving baby/ies | 13 | 0% | 1 (8%) | 12 (92%) | - Researcher: “Yes*, absolutely, I feel it’s really important, because you need to be able to discuss these risks with women, and to do that we need to be able to collect the information to have that discussion.”* |
| Pregnancy complications for the mother after a stillbirth is identified in a multiple pregnancy | 12 | 0% | 5 (42%) | 7 (58%) | - Parents discussed how they did not know how likely these risks were and so found it hard to vote on this outcome - Parent: *“I did lose my waters and continued for two further weeks, I wasn’t aware that there was a risk to me, only to the babies.”* - Discussion on whether this is covered by the “Life-threatening complications for mother” outcome in the main core outcome set |
| Neonatal outcomes of surviving baby(ies) after stillbirth is identified in a multiple pregnancy | 12 | 0% | 2 (17%) | 10 (83%) | - Discussed definition to include admission to intensive care unit, birth weight – final definition to be decided later in process |
| Neurodevelopment of surviving baby(ies) after stillbirth is identified in a multiple pregnancy | 12 | 0% | 6 (50%) | 6 (50%) | - Researcher *“feel like I’ve got a lot to say but maybe it’s because I actually manage these patients in the Foetal Medicine Unit a bit. But I feel like this is an absolutely key part of a discussion with a family about their babies or baby when we’re making decisions, and so I feel strongly that it’s really important, and can be very life altering for families and children. So I think it’s just one of the key things we end up actually discussing the parents that are interested in after survival.”* - Discussion whether to include in definition of “Neonatal outcomes” - Discussion on the feasibility of measuring this outcome in the long term - Parent: *“I agree I don’t want to see it lost, but I don’t really know where it fits, or the feasibility I think are quite big challenges.”* |
| Medical health of surviving baby | 12 | 0% | 9 (75%) | 3 (25%) | - Parents discuss how it is similar to the neonatal outcome previously voted on |
| Attachment to surviving baby | 12 | (2) 17% | 4 (33%) | 6 (50%) | - Discussion on how it is a long term outcome that could fall under mental health |
| Final vote and agreement on core outcome set | 12 | 0% | 0% | 100% |  |
